# Data-Driven Identification of Constraints and Enabling Pathways for Energy-Positive Wastewater Treatment

**DOI:** 10.1021/acs.est.6c01273

**Published:** 2026-06-03

**Authors:** Zi Zhang, Yuqing Yan, Xiatong Li, Emily Mayo, Zhiyong Jason Ren

**Affiliations:** † Department of Civil and Environmental Engineering, 6740 Princeton University, Princeton, New Jersey 08544, United States; ‡ Andlinger Center for Energy and the Environment, Princeton University, Princeton, New Jersey 08544, United States

**Keywords:** wastewater, net energy producer, intrinsic energy demand, carbon redirection, anammox, integrated modeling

## Abstract

The wastewater sector accounts for ∼1% of global electricity use, and demand will rise as countries expand treatment to meet Sustainable Development Goal 6. Transitioning wastewater treatment plants (WWTPs) from energy consumers to net producers is therefore crucial, yet the system-level feasibility of energy-positive operation remains unclear. Using a national inventory of 2961 WWTPs, we remove operational bias through Stochastic Frontier Analysis (existing in 70% of plants) and develop an efficiency-adjusted energy baseline. Energy balance modeling shows that, under conventional scenarios, energy-positive treatment is achievable only for high-strength wastewater (COD 334–426 mg/L, COD/TN ratio 8.5–11.9), excluding most global facilities. However, when evaluating two carbon-redirection pathways that integrate enhanced primary treatment with mainstream Anammox, we find such pathways can reduce entry thresholds to COD 153–198 mg/L and COD/TN 4.9–5.7, enabling low-strength systems to approach energy positivity. Medium-scale facilities are found to be the most favorable candidates. This characteristic-driven framework motivates future research and pilot demonstrations aimed at expanding the frontiers of energy-positive wastewater treatment.

## Graphical abstract

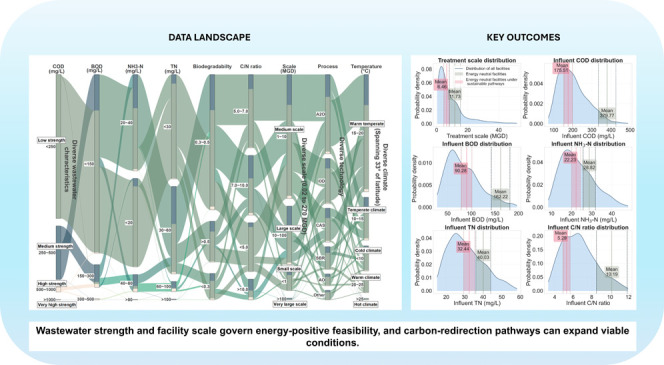

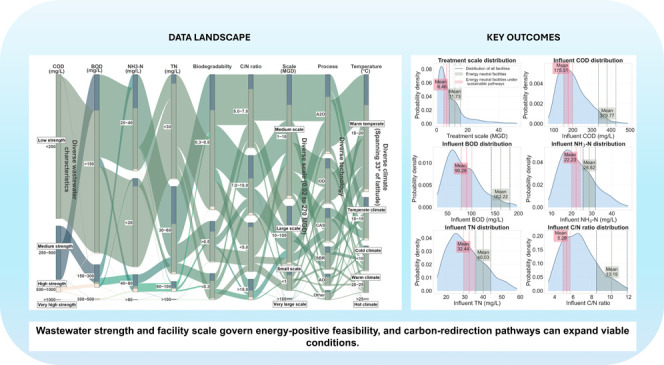

## Introduction

The global wastewater sector faces an important sustainability challenge: it is estimated to account for approximately 1% of global electricity consumption and significant carbon emissions, while basic service needs remain unmet in many regions.^1^ Approximately 3.6 billion people lack access to safe sanitation, and over 80% of wastewater is released untreated worldwide.^2^ Pursuing Sustainable Development Goal 6 by expanding through traditional treatment models would significantly increase energy demands, raising concerns about the long-term sustainability. This dilemma between service expansion and energy consumption is vividly illustrated in China. Since 2000, energy consumption for municipal wastewater treatment has more than tripled, with the annual treatment volume exceeding 70 billion m^3^ by 2021.^3^ Concurrently, energy use intensity (EUI) increased by over 20% between 2010 and 2018, largely driven by the implementation of more stringent discharge standards.^4^ These trends highlight a growing energy burden at the national level as the treatment coverage expands. Therefore, transitioning wastewater treatment plants (WWTPs) to become net energy producers presents a strategic opportunity, enabling expanded service coverage, deeper decarbonization, reduced operational costs, and enhanced resilience against grid and climate disruptions.^5,6^

Despite substantial progress, research on energy-positive wastewater treatment is fragmented, largely relying on site-specific, regional, and technology-specific case studies.^7–11^ This fragmentation is exacerbated by a limited availability of large-scale, high-quality operational data from full-scale treatment facilities, limiting the generalizability of findings and hindering a comprehensive understanding of energy dynamics across the sector. Existing methodologies often utilize simplified energy balances that overlook vital operational factors, such as actual biogas utilization efficiency and the climate-dependent energy demands of processes like anaerobic digestion.^12^ Furthermore, assessments of innovative treatment pathways, including influent carbon redirection and innovative biological nitrogen removal processes, are generally based on nominal flow characteristics, failing to adequately address systematic integration challenges or site-specific feasibility constraints imposed by real wastewater composition.^13,14^ Together, these limitations indicate the field still lacks a characteristic-driven, generally applicable framework that links wastewater composition, plant capacity, local climate, and process configuration to the realistic potential for energy-positive operation.

This study aims to address this research gap by analyzing a large national inventory of 2961 municipal WWTPs as a representative data set spanning broad gradients in treatment processes, plant size, wastewater characteristics, climatic conditions, discharge standards, sludge production, and EUI, making it a useful proxy for exploring patterns that are relevant to many global systems. However, deriving generally applicable insights from this extensive data set hinges on resolving a key methodological question: do the observed energy use data reflect the inherent energy requirements under optimal conditions, or are they skewed by facility-specific operational conditions? Previous studies have highlighted numerous operational challenges in full-scale WWTPs, underscoring the need to statistically isolate these effects to focus on the objective factors that govern inherent EUI.^15–17^ Establishing a technically efficient baseline, therefore, provides a more reliable foundation for assessing how wastewater characteristics, treatment processes, and climatic conditions shape energy demands.

Another important gap concerns the system-level energy implications of emerging treatment pathways. Conventional treatment faces an inherent “energy recovery ceiling”, particularly under low-strength wastewater,^18^ prompting increasing interest in “carbon-redirection” strategies that combine enhanced upstream carbon capture with mainstream Anammox processes to simultaneously increase biogas recovery and reduce aeration energy demand.^13,14,19–21^ Among these approaches, chemically enhanced primary treatment (CEPT) and high-rate activated sludge (HRAS) are two representative and widely studied technologies for upstream carbon capture through physicochemical and biological mechanisms, respectively, and both are broadly compatible with existing activated sludge infrastructure. Meanwhile, mainstream Anammox processes are increasingly recognized for their potential to substantially reduce aeration requirements and external carbon demand for nitrogen removal. Previous research indicates that redirecting an additional 10% of influent COD to energy recovery, in conjunction with mainstream Anammox processes that halve aeration energy, could significantly alter energy profiles.^22^ Accordingly, this study evaluates two integrated pathways: Pathway I combines CEPT with partial denitrification-Anammox (PdN/A), while Pathway II couples HRAS with partial nitrification-Anammox (PN/A).^14^ Assessing these systems requires integrated modeling, as pathway feasibility depends not only on theoretical energy balances but also on compatibility between carbon capture units and downstream nitrogen removal processes. Other carbon-redirection configurations, such as anaerobic membrane bioreactors, may also enable strong energy recovery, but they represent different treatment paradigms that are generally better suited to high-strength wastewater or sludge than to municipal wastewater. Their implementation also typically requires covered tanks and more substantial process modifications, along with different operational considerations such as membrane fouling, dissolved methane management, and overall system complexity.^23^

Taken together, this study makes three key contributions (Figure 1): first, by employing stochastic frontier analysis (SFA), we separate objective factors (treatment process, plant size, influent characteristics, effluent standards, and climate) from operational inefficiency, enabling us to approximate inherent energy requirements. Second, we develop an integrated kinetic process-energy modeling framework to evaluate both conventional processes and carbon redirection pathways under realistic influent and operational conditions. Third, we apply this framework to identify characteristic-based thresholds that determine where an energy-positive treatment is feasible. The analysis indicates that conventional treatment is constrained to high-strength wastewater conditions for achieving energy-positive operation, whereas carbon redirection strategies can substantially lower these barriers and expand feasibility, particularly for medium-scale facilities. Together, these insights provide a characteristic-driven basis for prioritizing where advanced treatment configurations can most effectively shift WWTPs from net-energy consumers to net-energy producers.

**Figure 1:**
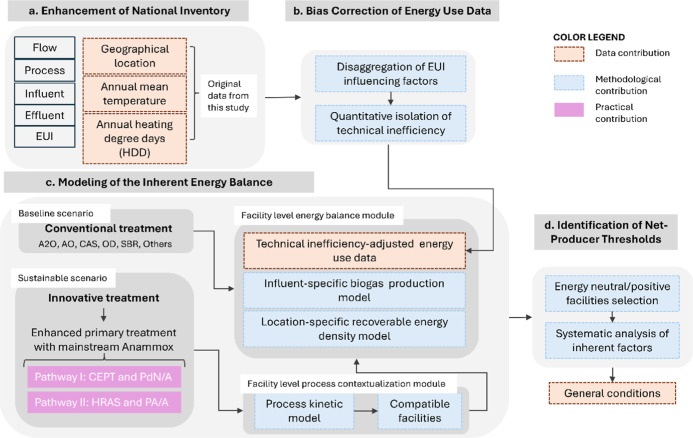
A four-phase methodological framework for identifying the inherent conditions enabling energy-positive wastewater treatment. The process encompasses national inventory compilation, bias correction via inefficiency isolation, inherent energy balance assessment, and the derivation of generalized conditions. Color legend indicates contributions from data (orange), methodology (blue), and practical insights (purple).

## Materials and Methods

This study uses a large, diverse national inventory of municipal WWTPs to establish the inherent conditions for energy-positive operations. We first applied statistical analysis and developed the stochastic frontier to disentangle technical inefficiencies from intrinsic energy performance, creating a bias-corrected benchmark data set. Two sustainable treatment scenarios—chemically enhanced primary treatment with mainstream Anammox (CEPT and PdN/A) and high-rate activated sludge with mainstream Anammox (HRAS and PN/A) are evaluated by integrating kinetic process models into facility-level energy balances. This coupled modeling approach ensures process compatibility and realistic energy profiling across real-world wastewater and climate conditions. Our analysis identifies generalizable conditions for energy-positive treatment, informing where advanced treatment configurations are most likely to enable energy-positive operation. The four-phase methodological framework supporting these findings is presented in Figure 1.

### Data Source

This study compiled wastewater treatment facility data from multiple national administrative data sets in China, including the Ministry of Ecology and Environment (providing plant specifications, permits, and technologies), the Ministry of Housing and Urban-Rural Development (containing wastewater characteristics and flow rate data), and the National Pollution Discharge Permit Management Information Platform (offering administrative records). The initial data sets comprised thousands of individual facility records, which were harmonized and merged using treatment volumes reported for 2019 (using a <0.1% discrepancy threshold). The final data set included 2961 georeferenced WWTPs, for which geographic coordinates were obtained via the Google Cloud Geocoding API. These coordinates allowed the extraction of location-specific climate variables, including annual average temperatures from NASA Power, and the calculation of Heating Degree Days (35 °C as a reference for anaerobic digestion).^24,25^ This climate data integration allowed for normalized energy consumption analysis and a more accurate assessment of energy recovery potential across different regions. The final data set includes secondary treatment processes (A2O, AO, CAS, OD, SBR, and others), while assuming standard operational boundaries of preliminary plus biological treatment only and 80% moisture content for sludge.^15^ All influent and effluent parameters (including COD, BOD, TN, TSS, and TP concentrations) were explicitly documented. Further details can be found in Supporting Information, Supplementary Methodology, Processing of the Data Set.

### Statistical Decomposition of Technical Inefficiencies

Previous research has reported operational and managerial challenges affecting full-scale WWTP performance.^16–18^ To derive generalizable insights, it was essential to distinguish subjective factors (equipment and managerial impacts) from objective factors (treatment process, scale, climate (represented by annual heating degree days, HDD), influent characteristics, and discharge standards). We initiated our analysis at the provincial level to reduce variability arising from facility-specific operational differences and measurement noise, thereby enabling more robust and comparable assessment across regions than would be possible using individual plant-level data. Treatment energy performance was evaluated using two metrics: flow-based EUI (kWh/m^3^) and COD removal EUI (kWh/kg COD-removed). Recognizing the limitations of either metric in isolation, we first normalized both metrics through *Z*-score transformation, then quantified interprovincial disparities on energy performance using pairwise distance analysis. We conducted Spearman’s correlation analysis to examine relationships between both EUI metrics and key objective parameters, including treatment scale, influent and effluent characteristics (COD, BOD, TN, NH_3_–N, TP, and TSS), and climatic variables. Concurrent ANOVA testing evaluated EUI variations across different treatment process configurations. We then applied principal component analysis (PCA) combined with *K*-means clustering to categorize provinces with similar objective parameters. To validate clustering robustness, we calculated coefficients of variation for within-cluster objective parameters, confirming homogeneous grouping (results in Table S18). This clustering enabled decomposition of total EUI variability into two components: between-cluster variation (attributable to objective characteristics) and within-cluster variation (reflecting subjective factors). Through ANOVA-based sum of squares decomposition, we quantified the relative contributions of each component as ratios of between-cluster (BSS) and within-cluster (WSS) sum of squares to total sum of squares (TSS) (eqs 1 and 2), providing quantitative insights into the fundamental drivers of EUI disparities across the national inventory (detailed Supporting Information, Supplementary Results Decomposition of Regional Energy Performance).

1 $$V_{between}=\frac{BSS}{TSS}$$

and

2 $$V_{within}=\frac{WSS}{TSS}$$

where *V*_between_ is the proportion of variation explained by between-group differences and *V*_within_ is the proportion explained by within-group differences.

### Stochastic Frontier Analysis

To statistically remove operational inefficiency from observed EUI, we applied SFA, which decomposes residuals into two distinct components: a stochastic error term (*v*) capturing random statistical noise and a technical inefficiency term (*u*) quantifying operational deficiencies.^26–28^ This dual-error structure makes SFA particularly well-suited for establishing efficiency frontiers and assessing energy performance in WWTPs.^27^ Prior to developing the SFA model, we assessed correlations between EUI and operational parameters (plant size, influent/effluent COD, BOD, TN, NH_3_–N, TSS, and TP) and conducted ANOVA on treatment processes. COD-removal EUI (kWh/kg COD-removed) was selected as the dependent variable because it exhibited consistently stronger correlations with key operational parameters than flow-based EUI, indicating greater sensitivity to treatment performance (Figure S3). While COD-removal-based EUI was used as the dependent variable for efficiency benchmarking, nitrogen-related energy demand was explicitly accounted for through the inclusion of influent TN and NH_3_–N as independent variables in the SFA model. ANOVA testing indicated no significant correlation between EUI and treatment process configurations (detailed results provided in Figure S3). Correlation analysis identified several variables demonstrating meaningful associations with EUI (|*r*| > 0.2), including treatment scale, influent COD, BOD, TN, NH_3_–N, TP, TSS, and effluent COD. These variables were subsequently incorporated as independent parameters in our SFA model, which employed a Cobb-Douglas cost function specification with half-normally distributed inefficiency terms and normally distributed random errors (eq 3). Parameters (β, σ_*u*_, and σ_*v*_) were estimated via maximum likelihood estimation, generating technical efficiency scores (EE_*i*_) bounded between 0 and 1, where 1 represents the frontier (best achievable) performance under comparable operating conditions, indicating that a facility operates with minimal inefficiency relative to its peers (eq 4).

To ensure robustness and reliability, we conducted comprehensive sensitivity and uncertainty analyses. We implemented a bootstrap resampling approach to iteratively estimate the SFA model, enabling assessment of the stability and precision of the derived efficiency scores. This method also allowed characterization of parameter variability across multiple estimations, providing confidence intervals for all model coefficients. Our sensitivity analysis examined the influence of outliers on model stability through systematic exclusion of extreme observations. The complete results of these rigorous validation procedures are presented in Tables S11 and S12.

3 $$\mathrm{Ln}\left(EUI_{i}\right)=\beta_{o}+\sum_{j=1}^{k}\beta_{j}\;\mathrm{Ln}\;x_{ji}+v_{i}{+u}_{i}$$

4 $$EE_{i}=e^{-\mu_{i}}$$

where EUI_*i*_ is the COD removal EUI of plant *i* (kWh/kg COD-removed), *x*_*ji*_ is the input *j* (treatment scale, influent COD, BOD, NH_3_–N, TN, TSS, TP, and effluent COD), *v*_*i*_ is the random noise (*v*_*i*_ ∼ *N*(0,*σ*_*v*_^2^)), and μ_*i*_ is the inefficiency term (*u*_*i*_ ∼ *N* ^+^(0,*σ*_*u*_^2^)).

### Integrated Kinetic Process and Energy Balance Modeling Framework

Comprehensive assessment of facility-level energy balance requires quantifying both efficiency-adjusted consumption and recoverable energy. The EUI used in this study was derived from reported plant data and primarily represents electricity consumption in the liquid treatment line (preliminary and biological processes) because plant-specific data for sludge treatment energy were not consistently available. Rather, solids processing was incorporated within the energy balance modeling framework, where sludge production, dewatering, and anaerobic digestion are represented using process-based assumptions to estimate both additional energy demand and recoverable energy. We applied a hybrid kinetic model to estimate recoverable energy density via mesophilic anaerobic digestion (35 °C), employing combined heat and power conversion efficiencies of 35% for electricity and 40% for thermal energy.^29^ A distinctive feature of our modeling approach is the incorporation of site-specific energy utilization patterns, which accounts for actual facility demands for both electricity and thermal energy rather than relying solely on theoretical wastewater energy content.^30^ This methodology establishes “recoverable energy density” as a realistic metric for achievable energy recovery (detailed in Supporting Information, Supplementary Methodology Baseline Energy Balance Modeling and Table S1). Model robustness was verified through sensitivity analysis of anaerobic digestion parameters, including waste utilization rate, biomass decay rate, and biomass yield coefficient, across literature-derived ranges to quantify methane production variability.^29^

To overcome limitations in conventional energy balance assessments, which were often constrained by nominal flow conditions and traditional treatment configurations, we employed actual influent characteristics from the national inventory to evaluate two innovative treatment pathways: (1) CEPT and PdN/A (Pathway I) and (2) HRAS and PN/A (Pathway II).^31,32^ In this study, Anammox was not explicitly modeled; instead, it was treated as a downstream constraint, with feasibility evaluated based on whether effluent characteristics from upstream processes meet established requirements (e.g., COD/TN < 2) for stable Anammox operation.^31,32^ Phosphorus removal was not explicitly modeled; for configurations such as HRAS + PN/A, additional treatment (e.g., chemical precipitation) would likely be required to meet discharge standards, whereas CEPT can provide simultaneous carbon capture and partial phosphorus removal. These pathways alter energy profiles by increasing carbon redirection to digestion, reducing aeration requirements, and introducing additional process-specific energy demands.

We recalibrated baseline EUI to reflect these modified consumption patterns, establishing new efficiency frontiers to derive unbiased EUI estimates. The sensitivity of these revised EUI values was assessed against key parameters including dewatering intensity (5–20 kWh/ton dry sludge), sludge solid content (±10%), and process-specific electricity use intensities (±10%). At the same time, kinetic modeling of carbon capture units (CEPT and HRAS) ensured operational compatibility with downstream mainstream Anammox processes while simultaneously quantifying carbon redirection for enhanced biogas production.^33–37^ Process model reliability was further validated through systematic sensitivity analyses: CEPT modeling evaluated removal rates of COD, BOD, TN, and suspended solids (±5% variation), along with coagulant dosage, sludge yield coefficients, and mixing energy intensity (±10% variation). The HRAS model sensitivity assessed hydraulic retention time, solids retention time (±50% variation), and influent fast-soluble biodegradable fraction (±5% variation), with outputs checked for effluent C/N ratio, sludge production, and COD redistribution (complete methodology of kinetic models in Supporting Information, Supplementary Methodology Integrated Process Modeling and Energy Balance Modeling of Innovative Pathways, with detailed parameters in Tables S2–S9; sensitivity assessment detailed in Supporting Information, Uncertainty Characterization and Sensitivity Assessment). The selected variation ranges were grounded in literature-reported operational and design values for CEPT and HRAS systems; for example, CEPT removal efficiencies (COD: 40–70%, BOD: 50–80%, TSS: 60–90%) and coagulant dosages (10–100 mg/L) are well documented, while HRAS systems typically operate at short HRT (0.5–2 h) and SRT (0.2–2 d).^33,34,38,39^ The applied perturbations (±5–50%) therefore represent sensitivity within realistic operational ranges rather than absolute bounds.

### Deriving General Conditions for Facilities of Net Energy Producer

Finally, we identified objective conditions enabling energy-positive treatment under conventional and innovative paradigms. First, facilities achieving energy-positive under conventional configurations were characterized by treatment scale, influent COD, BOD, TN, NH_3_–N concentrations, and COD/TN ratio. The parameter envelope for successful energy performance was quantified by calculating 95% confidence intervals for each parameter. Next, each facility’s compatibility with Pathway I or II was evaluated, and only facilities newly achieving energy positivity under innovative pathways were included in the “innovative cohort”. Their objective parameters were likewise summarized using 95% confidence intervals to enable direct comparison. This methodological approach enables quantitative assessment of how innovative technologies alter the fundamental conditions for net energy positive in wastewater treatment, providing generalizable insights into the objective parameters governing energy performance across treatment paradigms.

## Results and Discussion

### Landscape of Wastewater Treatment in National Inventory

Our national inventory of 2961 WWTPs located in China encompasses a notable diversity in treatment processes, climatic conditions, plant scales, influent characteristics, and discharge requirements (Figure 2a). This extensive heterogeneity establishes the data set as an effective natural laboratory for investigating global wastewater challenges. Among the facilities, the Anaerobic-Anoxic-Oxic (A2O) process was the most prevalent (*n* = 1141), followed by Oxidation Ditches (OD, *n* = 652). The plants operate across a broad spectrum of annual average temperatures, from cold to hot tropical climates (spanning 33° of latitude), and treatment scales ranging from less than 1 to over 100 million gallons per day (MGD), with warm temperate climates and medium-scale plants being most common. While most facilities receive low- to medium-strength influent (COD < 250 mg/L, *n* = 2070; COD ∼ 250–500 mg/L, *n* = 769), the data set includes a considerable number of plants treating high- and very high-strength wastewater. The wide range of influent COD concentration within our data set offers globally relevant insights. This broad distribution mirrors global patterns reported by UN-Habitat, where influent strength typically increases with economic development. For example, high-income cities like Paris often have COD above 400 mg/L, while lower-middle-income areas like Laos and Thailand typically show levels below 100 mg/L.^40^ Furthermore, the influent COD/TN ratio is distributed relatively evenly across a range from 5 to >10. This diversity establishes the data set not only as a regional snapshot but also as a representative microcosm for investigating global wastewater challenges and opportunities.

**Figure 2:**
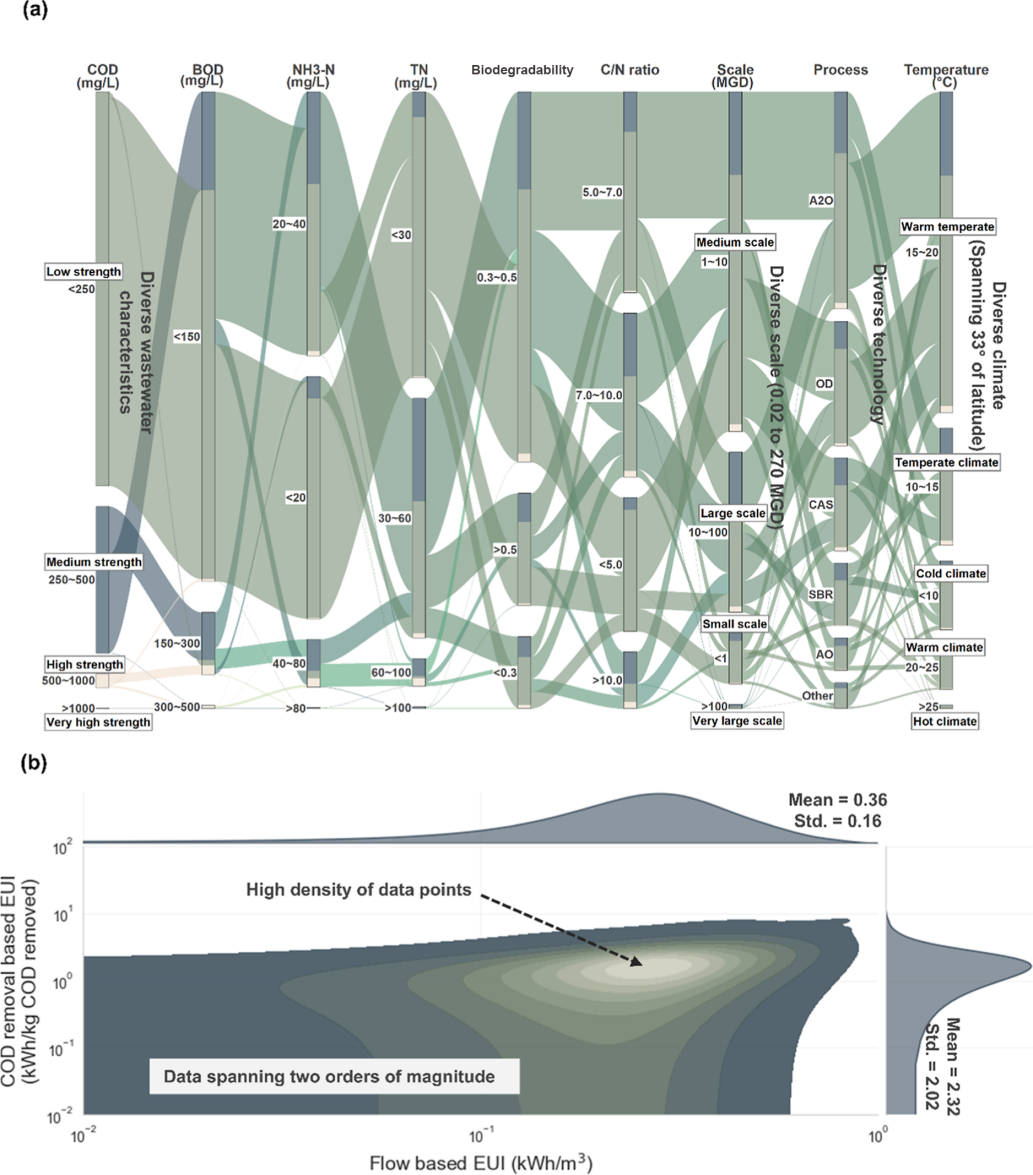
Diversity of wastewater treatment conditions and energy performance across 2961 facilities. (a) Inventory overview of 2961 wastewater treatment facilities, illustrating variability in influent characteristics, treatment scale (<1 to >100 MGD), process configurations (A^2^/O, AO, OD, CAS, SBR, and others), and climatic conditions (annual mean temperature ranging from −0.25 to 27 °C). Node and link colors are consistently mapped to influent strength categories; within each node, color proportions indicate the distribution of wastewater strength across facilities. (b) Joint distribution of flow-based and COD-removal-based EUI, spanning 2 orders of magnitude. Kernel density contours delineate the primary data cluster, while marginal histograms display the distributions of each metric.

A finding from this inventory is the high variability in EUI, which spans 2 orders of magnitude in both flow-based (kWh/m^3^) and COD-removal-based (kWh/kg COD-removed) metrics (Figure 2b). Such variability is consistent with earlier reports but is now demonstrated here at an unprecedented national scale.^17,41^ Interpreting EUI using both metrics is critical. For example, plants treating high-strength wastewater may appear efficient on a pollutant-removal basis yet still exhibit high flow-based EUI. This underscores the necessity of a dual-metric approach to avoid misclassification and to capture the full energy profile of a facility. Meanwhile, the magnitude of EUI variation raises a fundamental question central to benchmarking and optimization: what are the relative contributions of objective factors versus subjective factors? Objective factors include the biophysical and engineering context, including influent characteristics, climate, process selection, and discharge standards. They define the intrinsic energy demand. While subjective factors—including equipment condition, operational practices, operator performance and decision-making, and managerial strategies (hereafter referred to as technical inefficiency)—represent avoidable energy loss.^42^ Distinguishing between these two is methodologically essential. Many previous studies model raw EUI directly, successfully capturing real-world variability but inherently mixing intrinsic demand with inefficiency.^26,28,43,44^ Therefore, a model developed based on unadjusted EUI data would be biased, primarily capturing regional operational disparities rather than the fundamental biophysical relationships. Accordingly, a critical advancement of this study is the purification of national-scale energy data through statistical decomposition and SFA, enabling us to reveal the inherent energy requirements of wastewater treatment independent of technical inefficiency.

### Disentangling Technical Inefficiency from Energy Use Data

To derive generally applicable insights, it is essential to isolate the influence of technical inefficiencies from raw EUI observations. We first investigated the provincial-level disparities in EUI (Figure 3a). For instance, EUI in Shanghai and Gansu exhibit markedly different values, and these differences coincide with notable contrasts in influent COD concentrations (234 mg/L vs 150 mg/L). This raises a critical question for benchmarking: to what extent do observed EUI differences reflect inherent biophysical factors versus avoidable operational inefficiencies? We systematically evaluated the contribution of objective factors, including treatment process, climate, treatment scale, influent characteristics, and discharge standards, to EUI variability. An ANOVA test showed no statistically significant differences in EUI across treatment processes (Figure S3c,d), indicating that process selection alone cannot explain the national variation. Spearman correlations further showed that only a subset of parameters, including influent characteristics, treatment scale, and certain effluent standards, exhibited meaningful correlations with EUI (|*r*| > 0.2, *p* < 0.05 ^45^) for both flow-based and COD-removal-based EUI metrics (Figure S3a,b). These modest correlations suggest that objective factors alone are insufficient to account for the wide range of EUIs.

**Figure 3:**
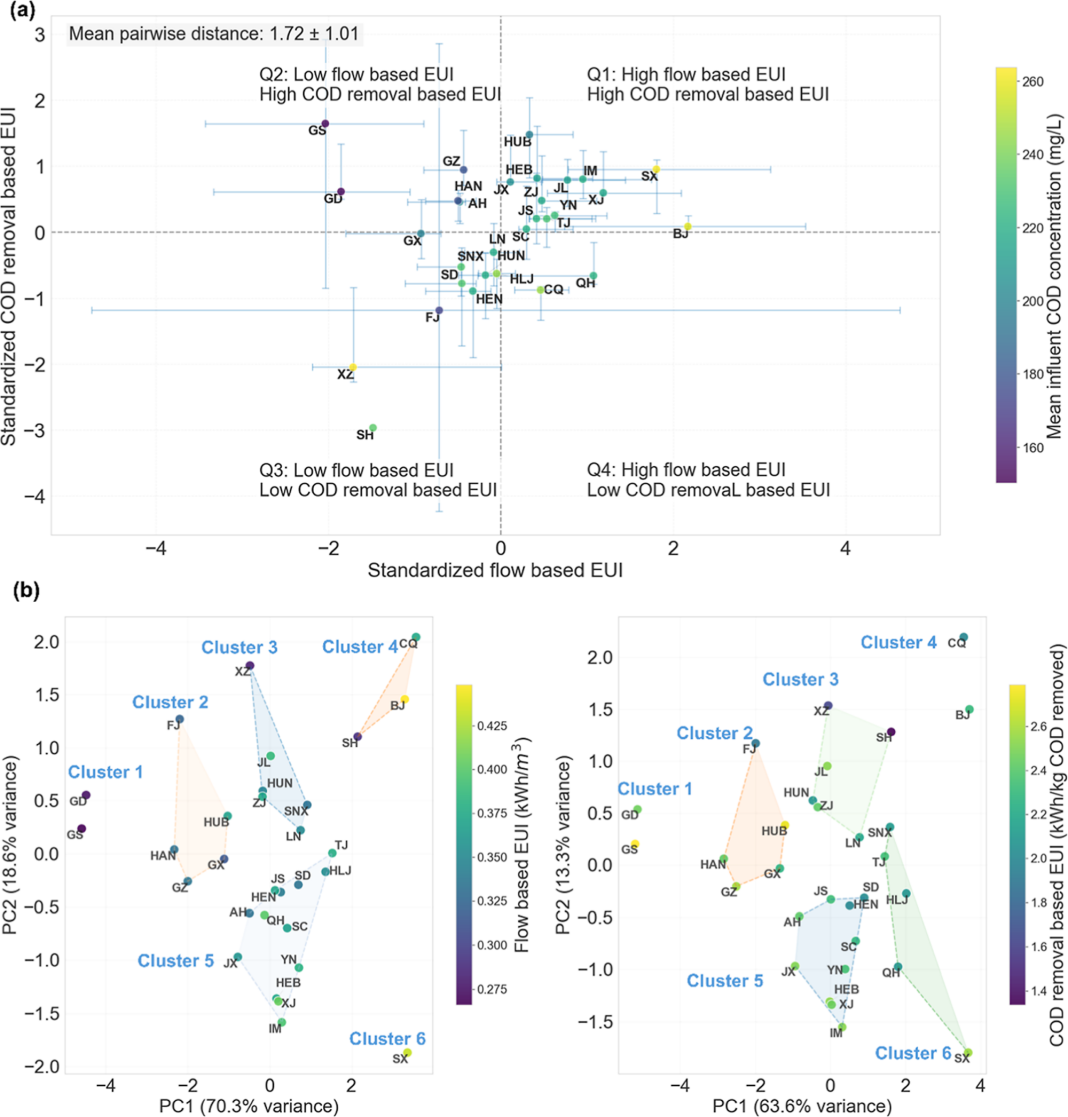
Regional disparities and objective-factor clustering in EUI. (a) Provincial comparison of flow-based (kWh/m^3^) and COD-removal-based (kWh/kg COD removed) EUI. The distance of each point from the origin (representing national means) quantifies regional disparities; both axes are *Z*-score normalized. (b) Unexplained EUI disparities per regional cluster from PCA. The analysis reveals six distinct regional clusters, each with highly uniform influent characteristics (<8% coefficient of variation), indicating that operational factors beyond influent quality are significant drivers of energy performance. (China’s provincial level acronyms are listed in Table S19; data for Ningxia, Hong Kong SAR, and Macau SAR were unavailable.)

To further decouple the influence of objective and subjective factors, we applied PCA for dimensionality reduction, followed by *K*-means clustering to group provinces with similar objective profiles.^46^ This resulted in clusters with high internal objective factors’ homogeneity (within-group coefficient of variation <8%, Table S18). Yet, even within these clusters, substantial residual variability in EUI persisted, 52% for flow-based and 72% for COD-removal-based EUI (Figure 3b and Table S10). For example, under similar objective conditions, Shanghai, Beijing, and Chongqing show divergent flow-based EUI values (0.29, 0.48, and 0.37 kWh/m^3^). Similarly, Jilin and Shanghai shared comparable objective profiles yet diverged in COD-removal-based EUI (1.30 vs 2.33 kWh/kg COD removed). These consistent within-cluster disparities, despite objective homogeneity, provide compelling evidence that technical inefficiency is a major and pervasive driver of energy performance variation. Such regional differences in inefficiency likely reflect variations in operational practices, operator expertise, facility age and equipment condition, and management capacity, which are not explicitly captured in the model but are inherently represented in the inefficiency term. Having established its significance, we proceed to formally quantify this inefficiency.

### Quantifying Technical Inefficiency and Developing Non-Biased Energy Use Baseline

Our previous analysis established that subjective factors significantly influence EUI, as evidenced by the considerable EUI variation among facilities treating similar wastewater. SFA model robustness was verified through rigorous uncertainty and sensitivity analyses, including 500 bootstrap iterations, which yielded a low variation in efficiency scores (2.4% ± 3.7%). Potential outliers affecting both regression parameters and efficiency scores were evaluated and addressed prior to final model construction (Tables S11 and S12). The SFA results revealed that the systematic inefficiency term (μ) was six times larger than the random noise term (ν), indicating that technical inefficiency, not chance, is the primary driver of excess energy use. The model estimates that the average facility operates at 63% of its technical potential. Notably, 70% of facilities have an efficiency score below 1, suggesting widespread potential for operational improvement; many have the potential to nearly halve their energy use to match their best-performing peers (Figure 4a). Spatial patterns further showed unequal distribution of efficiency, as shown in Figure 4b. Southeast provinces like Fujian and Guangdong exhibit higher efficiency, with 67% and 66% of facilities classified as efficient, respectively. In contrast, Northeast and North provinces perform poorly, typically with fewer than 30% efficient facilities, creating a clear geographic concentration of inefficiency. Finally, we corrected the raw EUI data by removing the estimated technical inefficiency. This adjustment, illustrated in Figure 4c, reveals the underlying energy demand under efficient operation. For flow-based EUI, the mean decreased from 0.35 kWh/m^3^ to 0.19 kWh/m^3^, and the data range narrowed significantly, indicating reduced heterogeneity. Similarly, the mean for COD-removal-based EUI fell from 2.32 to 1.18 kWh/kg COD, providing a benchmark for the sector’s energy-saving potential.

**Figure 4:**
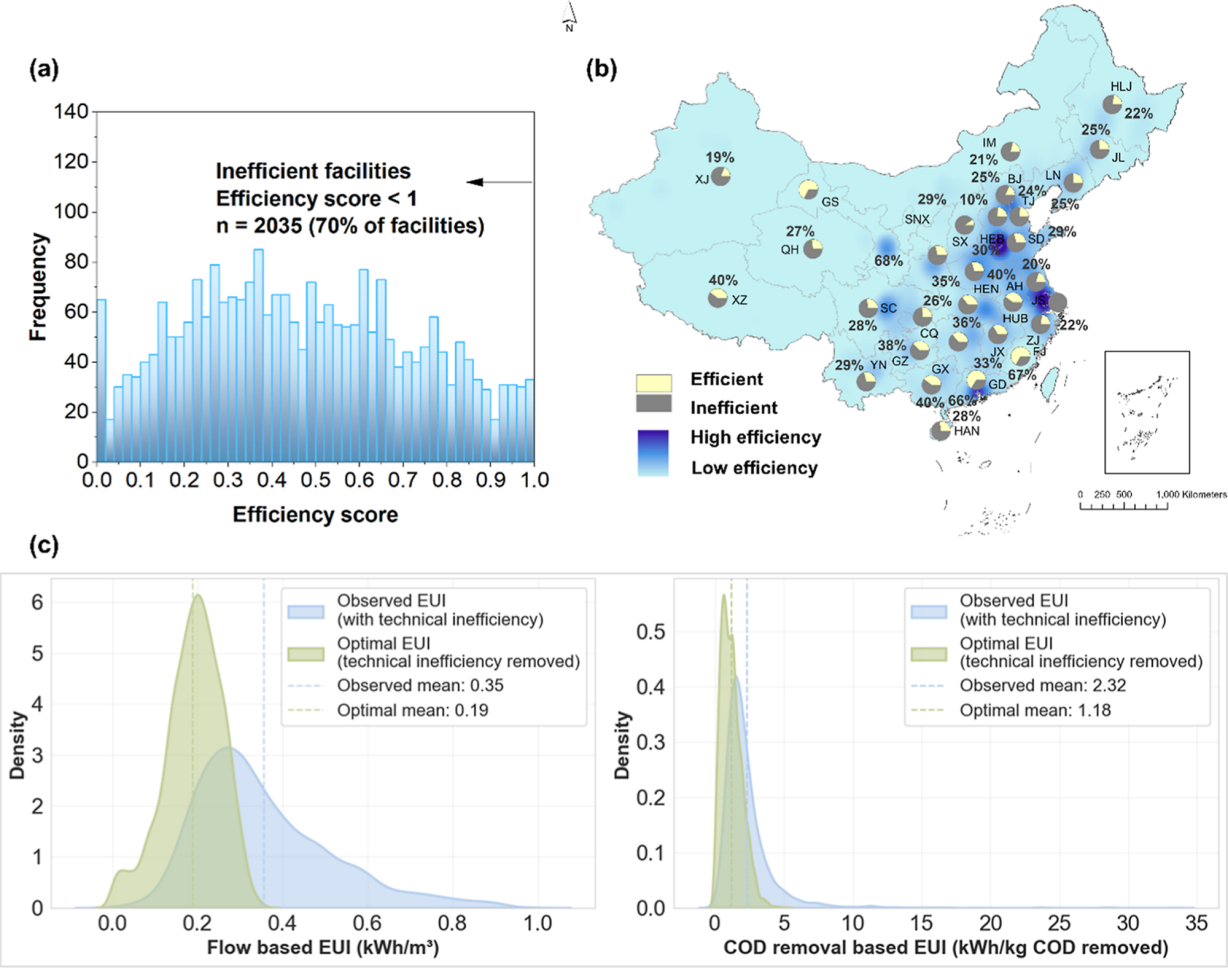
Impact of technical inefficiency on wastewater treatment energy performance. (a) The SFA score distribution defines facilities as efficient (score = 1) or inefficient (score < 1). (b) Spatial distribution of provincial operational efficiency. The intensity of blue indicates the concentration of highly efficient facilities, while pie charts show the proportion classified as efficient based on (a). (c) Potential energy savings from eliminating inefficiency, comparing the observed EUI distributions against the calculated optimal EUI for both flow-based (left) and COD-removal-based (right) metrics. Removal of technical inefficiency substantially lowers mean EUI and narrows variability.

### Systemic Barriers to Energy-Positive Wastewater Treatment

The transformation of WWTPs from energy consumers to net energy producers represents an appealing proposition. Yet the extent to which existing treatment configurations can truly deliver energy-positive performance remains poorly defined at a system level. In this study, the effective recoverable energy density was derived from a hybrid kinetic biogas generation model for anaerobic digestion, using site-specific BOD removal as the primary input. A comprehensive sensitivity analysis identified waste utilization efficiency as the dominant model parameter, with a tested range of 0.6 to 0.9 resulting in a 40% variation in biogas yield (Table S13). Consequently, the value of 0.8 was selected to represent reasonably well-operated mesophilic digestion systems while still remaining below the upper bound reported in the literature-reported range. These findings reveal a fundamental inherent energy deficit (Figure 5a): the mean recoverable energy density (0.05 ± 0.03 kWh/m^3^) falls substantially below the efficiency-adjusted energy demand (0.19 ± 0.07 kWh/m^3^). This imbalance confines energy-positive status to a limited subset of facilities: fewer than 5% (138 of 2961) operate as net energy producers, while 90% of plants demonstrate below 50% energy self-sufficiency. These energy-positive facilities are predominantly medium- to large-scale plants and are largely associated with conventional secondary treatment processes, such as A2O, CAS, and OD systems. Sludge processing was assumed to be consistent across facilities (mechanical dewatering followed by mesophilic anaerobic digestion), indicating that differences in energy performance are not driven by sludge treatment configurations in this analysis.

**Figure 5:**
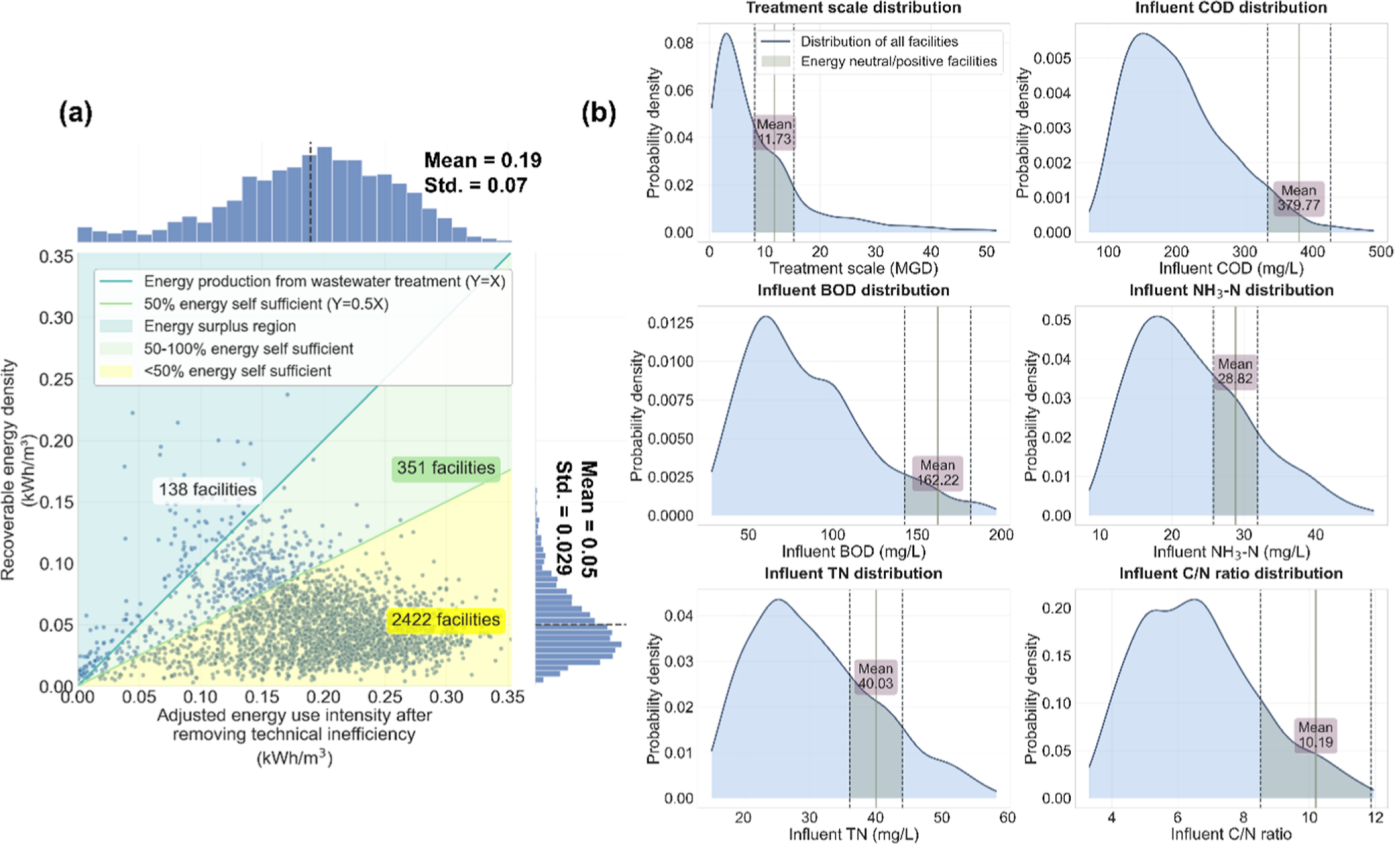
Energy balance and inherent conditions for energy-positive operation under the conventional treatment paradigm. (a) Facility-level comparison of optimal EUI (after removing operational inefficiency) versus recoverable energy density in wastewater. The shaded bands classify facilities as energy-positive (light blue), ≥50% self-sufficient (light green), and <50% self-sufficient (light yellow). (b) Influent characteristics of energy-positive facilities. The distributions (shaded) represent the full national data set for each parameter, while the dashed lines indicate the 95% confidence interval for conditions that achieve energy production.

Examination of the conditions enabling energy positivity identified two critical prerequisites (Figure 5b). First, facilities associated with energy-positive performance tend to fall within a narrow scale window (95% CI: 8.16–15.3 MGD; mean: 12 MGD). This pattern likely reflects scale-dependent trade-offs, where smaller plants may lack economies of scale for efficient energy recovery, whereas larger plants may experience energy demands that outpace recoverable energy. Second, energy-positive operation requires high-strength wastewater, characterized by elevated influent COD concentrations (95% CI: 334–426 mg/L; mean: 380 mg/L) and COD/TN ratios (95% CI: 8.5–11.9; mean: 10.2). These parameters establish a structural entry threshold that systematically excludes a significant number of facilities worldwide, particularly those treating low-strength wastewater. Threshold values of the COD/TN ratio reflect the combined influence of carbon availability and nitrogen loading, highlighting the critical role of the COD/TN ratio in determining both aeration energy demand and overall energy balance. Together, these findings demonstrate that current wastewater treatment and energy recovery paradigms impose structural barriers that limit widespread adoption of energy-positive operation. The identification of restrictive concentration thresholds indicates that incremental improvements or marginal process optimizations are insufficient to achieve widespread energy production. Instead, overcoming this inherent energy deficit will require fundamental technological innovations that lower entry barriers, particularly for facilities processing low-strength wastewater, which represents the majority of global systems.

### New Treatment Pathways Lower Structural Barriers for Energy-Positive Treatment

Achieving energy-positive wastewater treatment requires coordinated efforts to enhance recoverable energy density while reducing energy use intensity.^30^ Among various emerging approaches, carbon redirection strategies that combine upstream carbon capture with reduced aeration demand have been proposed as promising pathways to lower the barriers for energy positivity. In this study, we focus on configurations that integrate enhanced primary treatment with a mainstream Anammox as representative examples. Specifically, we evaluated two specific pathways: CEPT with PdN/A as Pathway I and HRAS with PN/A as Pathway II (Figure 6a). Unlike previous studies that assumed nominal compatibility, we developed integrated kinetic models to assess whether effluents from carbon capture units meet the biochemical requirements (COD/TN ratio <2) for downstream Anammox.^20,31,32,47^ Compatibility assessment revealed substantial variation across climatic and influent conditions (Figure 6b), with Pathway I exhibiting broader compatibility, particularly in southern provinces including Fujian, Guangdong, Guangxi, Guizhou, Yunnan, and Hainan, where over 50% of facilities are suitable. In contrast, Pathway II shows higher applicability in northern regions such as Tianjin and Shanxi, with approximately 30% of facility compatibility.

**Figure 6:**
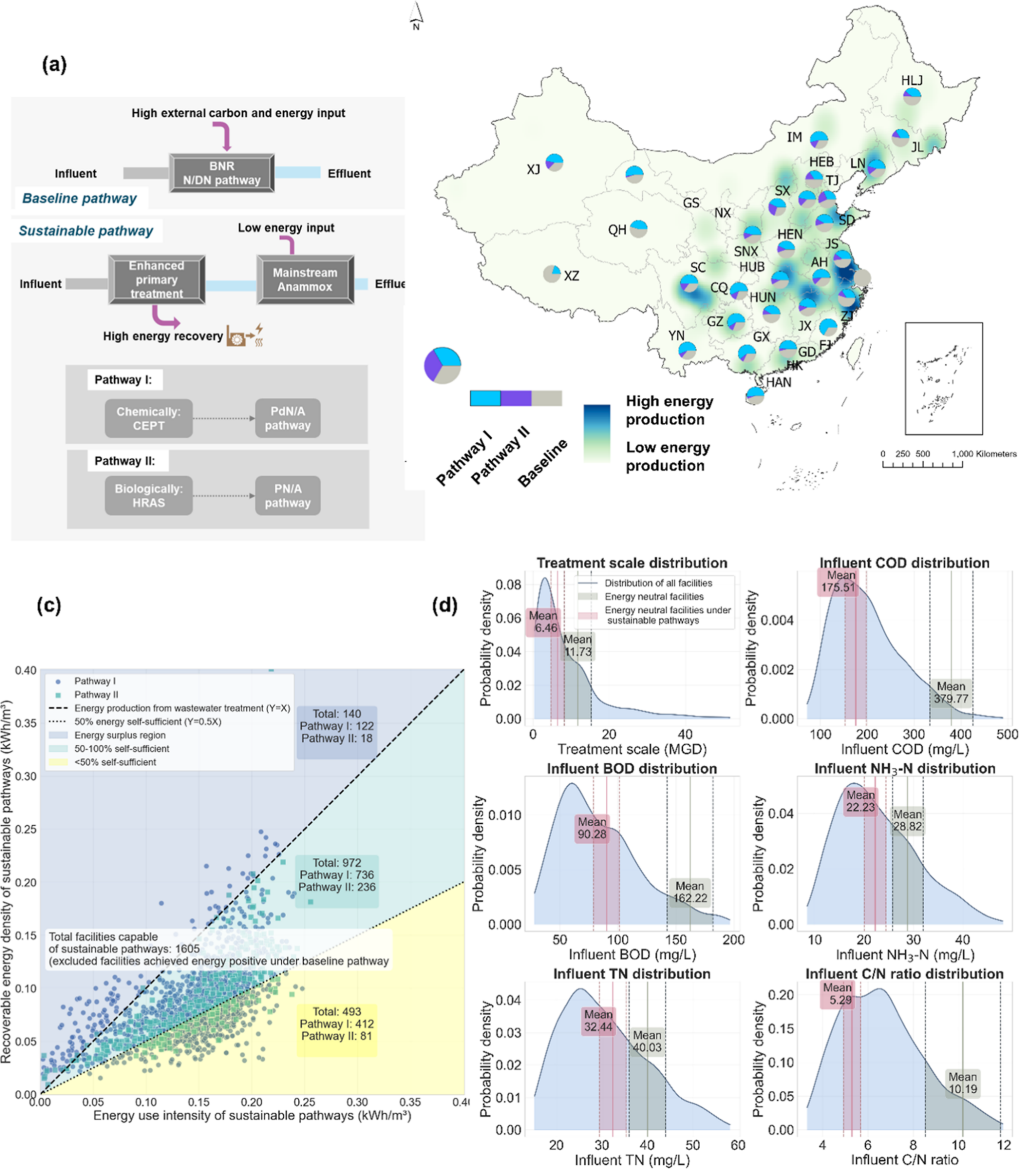
Compatibility and energy potential of sustainable wastewater treatment pathways. (a) Schematic comparison of the conventional baseline against two carbon-redirection pathways: CEPT (Pathway I) and biologically enhanced primary treatment (Pathway II), both integrated with mainstream Anammox. (b) Spatial assessment of pathway compatibility. Pie charts show the provincial proportion of facilities suitable for each pathway, while the blue intensity indicates the magnitude of potential energy production from wastewater. (c) Adjusted EUI versus recoverable energy density for the 1605 facilities capable of implementing Pathways I and II (facilities already energy-positive under the current paradigm are excluded). (d) Expansion of feasible influent conditions. The influent characteristics for achieving energy-positive are shown, with the red dashed line and shaded area indicating how sustainable pathways relax the constraints compared to the current.

To quantify system-level energy implications, we modified our energy balance model to incorporate carbon redistribution effects. Both CEPT and HRAS redirect organic carbon to sludge streams, enhancing biogas production while modifying dewatering and heating energy demands. Simultaneously, Anammox-based nitrogen removal substantially reduces aeration energy, but CEPT and HRAS increase energy demand.^14^ Through sensitivity analyses, we identified the COD removal rate for CEPT and sludge retention time for HRAS as the most critical parameters governing process performance (Tables S14–S17). Integrated energy balance modeling demonstrates that these pathways enable 140 additional facilities to achieve energy positivity, 122 through Pathway I and 18 through Pathway II, while nearly 40% of all facilities (972 plants) could exceed 50% energy self-sufficiency (Figure 6c). Most importantly, these pathways dramatically lower entry barriers for energy production (Figure 6d). The viable influent COD threshold decreases from 380 mg/L under conventional treatment to 153–198 mg/L (mean 176 mg/L) under sustainable pathways, while the COD/TN ratio threshold drops from 8.5–11.9 to 4.9–5.7 (mean 5.3). This shift represents a paradigm change: facilities treating low-strength wastewater, which were previously excluded under conventional paradigms, can now realistically approach or achieve energy-positive operation.

Notably, the two pathways exhibit distinct applicabilities across facility types (Figure S9). Pathway I (CEPT + PdN/A) is more broadly applicable, particularly for facilities with lower influent strength and existing A2O, AO, or OD configurations, whereas Pathway II (HRAS + PN/A) is better suited to facilities treating higher-strength wastewater and equipped with CAS or SBR systems. This highlights that pathway selection should be condition-specific, depending on influent characteristics, infrastructure, and local conditions. Geographically, the highest energy production potential concentrates in southeastern coastal regions, aligning with areas exhibiting superior pathway compatibility. Overall, these results establish that sustainable wastewater treatment requires context-specific technological integration. The identified pathways overcome fundamental limitations of current treatment paradigms by dramatically reducing the concentration and operational thresholds. This redefines the global potential for energy recovery in wastewater treatment, demonstrating that strategic process integration can transform diverse facilities, including those handling low-strength streams, from energy sinks to renewable energy sources.

## Implications

This study demonstrates that the energy-positive operation of wastewater treatment is not a distant ideal but an attainable outcome under the right conditions. Our findings reveal that its feasibility is shaped by fundamental constraints in wastewater characteristics, plant scale, and current treatment processes, underscoring the need for a realistic and evidence-based roadmap. By establishing a bias-corrected, national-scale energy baseline, we show that the conventional pathway supports energy-positive performance only for a subset of facilities treating high-strength wastewater, a finding that tempers overly optimistic projections and clarifies where today’s technologies can truly succeed. The true paradigm shift emerges from integrating enhanced primary treatment with mainstream Anammox, which can lower the COD threshold for energy-positive operation, expanding the pool of globally viable facilities. Our analysis identifies medium-scale facilities as the optimal candidates for this transition under either pathway.

Meanwhile, we want to note that energy recovery estimates depend on assumptions regarding digester performance, waste utilization efficiency, biogas-to-energy conversion, and sludge management practices, all of which vary across climates, operational setups, and regulatory contexts. Similarly, the long-term stability of mainstream Anammox under real-world fluctuations in temperature, load, and inhibitory compounds remains an active area of research. Future work should incorporate dynamic, time-series operational data, technoeconomic assessments, and life-cycle analyses to better quantify these uncertainties and evaluate trade-offs among energy, emissions, and cost. Integration with emerging technologies, such as electrochemical carbon concentration, AI-guided process control, advanced sludge pretreatment, and distributed energy storage, may further shift the feasibility boundaries.

Overall, this study provides a characteristic-driven, generalizable framework that can guide utilities, policymakers, and technology developers on where and how to prioritize next-generation treatment trains. By linking influent quality, plant scale, climate, and process configuration to realistic energy-positive potential, we highlight a pathway for transforming wastewater treatment from a linear energy consumer into a flexible, distributed renewable energy node within the urban water-energy-climate nexus.

## Supplementary Material



## Data Availability

All data processing, analyses, and visualizations for this study were performed using Python 3.12. The newly compiled national WWTP inventory, along with the derived data sets on process performance and energy profiles, is publicly available. The complete code used for data processing, analysis, and figure generation is also provided in our GitHub repository: https://github.com/zzhanger/Energy-neutral-positive-wastewater-treatment.git.

## Notes

The Supplementary Information includes detailed descriptions of the dataset compilation and validation procedures, assumptions and methodologies used in the energy balance and process modeling framework. It also provides parameter tables, sensitivity and uncertainty analyses, stochastic frontier analysis details, additional regional energy performance results, and supplementary figures and tables supporting the main manuscript findings (PDF)
