# Supplementary material for: Data-Driven Identification of Constraints and Enabling Pathways for Energy-Positive Wastewater Treatment
Source: Environ Sci Technol. 2026 Jun 3;60(23):16566–78. doi: 10.1021/acs.est.6c01273 (PMC13276910; doi:10.1021/acs.est.6c01273)
Supplement: Supplementary file 1 [file es6c01273_si_001.pdf]

Supplementary information for:

Data-driven identification of constraints and enabling pathways for energy-positive wastewater treatment

Authors

Zi Zhang[1,2], Yuqing Yan[1,2], Xiatong Li[1,2], Emily Mayo[1,2], Zhiyong Jason Ren[1,2*]

Affiliations

[1]Department of Civil and Environmental Engineering, Princeton University, Princeton, NJ 08544, USA

[2]Andlinger Center for Energy and the Environment, Princeton University, Princeton, NJ 08544, USA

[*] Correspondence: zjren@princeton.edu

This supplementary information includes:

Supplementary methodology

Supplementary results

Table S1 to S19

Figures S1 to S9

SUPPLEMENTARY METHODOLOGY

Processing of the dataset

Data sources:

The data used in this study was obtained from three government sources: 1) the Ministry of Ecology and Environment (MEE) (dataset 1), 2) Ministry of Housing and Urban-Rural Development (MOHURD) Office (dataset 2), and 3) The National Pollution Discharge Permit Management Information Platform (NPDPMIP) (dataset 3). The first data source from the MEE contained information on the treatment plant name, discharge permit, treatment technology, text location, design capacity, and actual flow rate. The second data source from the MOHURD Office provided details on the influent and effluent water quality parameters as well as the actual flow volumes in 2019. The third source was accessed and compiled from an online open-source platform, which consists of the plant name and discharge permit.

Original data processing:

The original dataset 1, dataset 2, and dataset 3 contained 9,221, 5,157, and 16,663 records of wastewater treatment plants (WWTPs) in China, respectively. Upon further investigation, it was found that some records in dataset 1 had discharge permits that could not be retrieved from the NPDPMIP. These records were regarded invalid, and dataset 3 was used to validate dataset 1 to ensure that all WWTPs in the final dataset had valid discharge permits. After this validation process, a total of 5,462 records were retained, representing the wastewater treatment plants with valid discharge permits (in normal operation). The next step was to combine this valid dataset with dataset 2, using the treatment volume data from 2019. A preliminary check revealed that the annual treatment volume values in dataset 1 and dataset 2 varied by less than 0.1%, likely due to minor human errors during recording. For example, annual treatment volume values of 1,862.73 (10,000 tons) and 1,862.75 (10,000 tons) in dataset 1 and dataset 2, respectively, likely belonged to the same wastewater treatment plant. Using a 0.1% threshold for the difference in treatment volume in 2019 between the valid dataset and dataset 2, the integrated dataset was compiled, containing information from both dataset 1 and dataset 2. This integrated dataset ensured that the included WWTPs were currently in operation and had valid discharge permits, resulting in a total of 3,989 records.

After compiling the integrated dataset, we utilized the Google Cloud Geocoding API to obtain the geospatial data (longitude and latitude coordinates) for each WWTP. This was achieved by providing the Province, City, and text location as the key parameters to retrieve the corresponding geospatial coordinates. The geospatial data for all WWTPs were then visualized using ArcGIS Pro software (shown in Figure S1). To validate the accuracy of the acquired spatial data obtained through geocoding, a random sampling method was employed. This allowed us to cross-check the precision of the longitude and latitude coordinates for a subset of WWTPs. During this validation process, we noticed that some of the geospatial information was inaccurate. To address this, we

conducted a spatial join operation between the province boundary layer and the WWTP location layer. This analytical technique enabled us to cross-reference the province attribute associated with each WWTP's geographic coordinates against the actual province boundary within which the treatment plant was situated. Any WWTP records where the joined province did not align with the originally attributed province were flagged as containing inaccurate location data. These erroneous entries were then removed from the final dataset to ensure the integrity and reliability of the WWTP geospatial information for subsequent analysis and visualization

Assumptions for the dataset

The compiled dataset comprises six categories of secondary treatment processes: anaerobic/anoxic/aerobic (A2O, n=1,141), anoxic/aerobic (AO, n=171), conventional activated sludge (CAS, n=489), oxidation ditch (OD, n=652), sequencing batch reactor (SBR, n=329), and others (n=139). Based on the limited use of primary settling tanks in China, we assumed all facilities in the dataset employ only preliminary treatment followed by secondary biological treatment. Regarding energy use intensity (EUI), the dataset did not specify system boundaries for energy reporting, as China lacks national regulations standardizing wastewater treatment energy assessment boundaries. While Guangdong province established Energy Consumption Standards for Municipal Sewage Treatment in 2020 defining comprehensive boundaries (including preliminary treatment, biological treatment, advanced treatment, disinfection, sludge dewatering, and auxiliary systems), this regulation remains geographically restricted[1]. Consequently, we assumed the reported EUI values reflect only preliminary and biological treatment processes. For sludge data, since the original dataset did not indicate moisture content, we compared the reported annual sludge yields with national statistics (typically reporting 80% water content sludge) and found them comparable in magnitude, leading us to assume 80% water content for all sludge data[2]. All other parameters - including average flow rates and influent/effluent concentrations of COD, BOD, SS, NH_3-N, TN, and TP - were clearly defined in the dataset.

Framework for integrated process modeling and energy balance modeling

Baseline energy balance modeling

We developed a comprehensive energy balance model integrating both energy consumption (Equation S1) and energy recovery (Equation S2) components. Given China's current limited energy recovery practices (with anaerobic digestion (AD) adoption below 5%)[3], we specifically focused on AD with combined heat and power (CHP) as the primary energy recovery pathway. For energy consumption modeling, we utilized the dataset's energy use intensity (EUI) values to represent electricity consumption for preliminary and biological treatment processes. Based on literature values, we allocated 60% of total electricity consumption to aeration processes and 40% to other operational needs (sensitivity analysis was conducted)[4]. Sludge dewatering was assumed to employ mechanical methods as the most prevalent technology[5]. The AD implementation would

introduce additional thermal requirements. We incorporated heating demands for maintaining mesophilic conditions in digesters (35℃), calculating location-specific heat consumption using annual heating degree days (HDDs) retrieved from NASA's POWER database. This thermal energy requirement was explicitly included in our energy consumption model. Influent carbon sources are partitioned among three pathways: effluent discharge, mineralization to CO_2, and incorporation into waste sludge. For energy recovery potential, we employed a hybrid kinetic methane generation model to estimate biogas production[6]. The resulting methane yield was then applied to CHP systems to determine recoverable electricity and thermal energy. Table S1 summarizes the parameters used in baseline energy balance modeling.

$$ED_i = ED_{elec_i} + ED_{heati} \quad \text{(Equation S1)}$$

$$ED_{elei} = EUI_i \cdot Q_i + e_{dewater} \cdot m_i \cdot c_{solid} \quad \text{(Equation S1-1)}$$

$$ED_{\mathrm{heat}i} = (1 + R_{loss}) \cdot m_i \cdot C_{sludge} \cdot 1000 \cdot \frac{HDD_{mesoi}}{3600} \quad \text{(Equation S1-2)}$$

$$ER_{(elec/\mathrm{heat})i} = \eta_{(power/\mathrm{heat})} \cdot \mu_{meth} \cdot \left(E \cdot S_{0_i} - F_{cell} \cdot \frac{\left(Y \cdot E \cdot S_{0_i}\right)}{1 + k_d \cdot \theta_c}\right) \cdot \sigma_{methane} \quad \text{(Equation S2)}$$

$$S_{0_i} = \left(BOD_{in_i} - BOD_{outi}\right) \cdot \theta_{aerobic} \cdot \frac{Q_i}{1000} * R_{BODL} \quad \text{(Equation S2-1)}$$

Where ED_i represents the total annual energy consumption of facility i, comprising both electrical ED_{eleci} and thermal components ED_{heati}, with ED_{eleci} accounting for energy use in both water and sludge processing lines and ED_{heati} representing site-specific heating requirements (primarily for digester heat maintenance); and where S_{oi} denotes the ultimate BOD of the influent sludge (kg/day), serving as the basis for calculating energy recovery potential from the system; all other parameters used in model were summarized in Table S1.

Table S1 Parameters used in energy consumption and energy recovery modeling in baseline wastewater treatment.

Parameter	For	Description	Source and value	Sensitivity check
$\boldsymbol{EUI}$	Energy consumption model	Energy use intensity of facility	From dataset	-
$\boldsymbol{m}$		Annual sludge yield (80% moisture)	From dataset	-
$\boldsymbol{Q}$		Annual treatment volume	From dataset	-
$\boldsymbol{e_{dewater}}$		Mechanical sludge dewatering energy use intensity [5]	5~20 kWh/tone dry sludge	Lower and upper bound

c_{solid}		Sludge solid content [2]	20%	
R_{loss}		Heat loss of anaerobic digestor [7]	20%	±10%
C_{sludge}		Heat capacity of sludge	4.18 kJ/(kg*°C)	-
HDD_{mesoi}		Annual heating degree days to achieve mesophilic AD condition	From dataset (calculated from the NASA power data)	-
$BOD_{in,out}$	Energy recovery model	Influent and effluent BOD	From dataset	-
$\eta_{power/heat}$		Efficiency of power and heat output of typical combined heat and power unit [8]	35% for electricity and 40% for heat	±5%
μ_{meth}		Methane conversion from BOD	$0.35 m^3/kg\ BOD$	-
E		Efficiency of waste utilization [6]	0.6~0.9	Lower and upper bound
$\theta_{aerobic}$		BOD fraction for anabolism in aerobic system [9]	1/3	-
R_{BODL}		Ratio of 5-day BOD to ultimate BOD [6]	0.68	-
F_{cell}		Conversion factor of cellular material into BOD[6]	1.42 $gBOD/gVSS$	-
Y		Sludge yield[6]	0.04~0.1 $gVSS/gBOD$	Lower and upper bound
k_d		Endogenous coefficient[6]	0.02~0.04 d^{-1}	Lower and upper bound
θ_c		Sludge SRT[6]	20 days	-
$\sigma_{methane}$		Heat value of methane[10]	9.94 kWh/m^3	-

<u>Integrated process modeling and energy balance modeling of carbon redistribution pathways</u>

Part I: Process modeling for CEPT and HRAS

Current wastewater treatment in China predominantly utilizes conventional nitrification/denitrification (NDN) pathways for biological nitrogen removal (BNR), implemented through processes such as AO, A2O, and SBR. However, this pathway encounters significant challenges due to the typically low COD/TN ratios in Chinese wastewater, averaging 6.9, with 43% of facilities reporting ratios below 6 (see Figure S2). This often requires external carbon supplementation to meet the necessary 6-10 g COD/g TN for complete heterotrophic denitrification[11]. Additionally, the nitrification process demands substantial aeration energy. Emerging autotrophic nitrogen removal pathways, particularly partial nitrification/anammox (PN/A) and partial denitrification/anammox (PdN/A), present promising alternatives for low C/N wastewaters. PN/A halts nitrification at the nitrite stage, reducing aeration energy by 60%[12]. Similarly, PdN/A requires only half the flow for complete nitrification, cutting aeration energy

needs by 50%[13]. Both pathways require influents with low C/N ratios, typically below 2[14,15]. To fully capitalize on these benefits, integrated systems that combine enhanced primary treatment methods—such as CEPT or HRAS—with Anammox processes can strategically redistribute carbon flows. The CEPT-PdN/A (pathway I) and HRAS-PN/A (pathway II) pathways show particular promise, capturing chemical energy in sludge while simultaneously reducing energy consumption. This approach represents a significant shift from conventional NDN methods toward more sustainable and energy-efficient wastewater treatment solutions.

The effective implementation of these innovative nitrogen removal pathways relies on the efficiency of primary treatment processes to achieve optimal C/N ratios for subsequent Anammox systems. To systematically assess this, we integrated complementary modeling approaches: empirical performance models for CEPT and modified ASM1-based kinetic models HRAS systems. Extensive studies confirm CEPT's consistent performance across diverse influent conditions (low COD <250 mg/L, medium 250-500 mg/L, high >500 mg/L), with empirical models reliably predicting effluent quality independent of loading characteristics[16–20]. Our literature-analysis of operational data (Table S2) demonstrates CEPT's physicochemical nature, while its model incorporates both TSS removal and coagulant effects (Table S3) to accurately estimate anaerobic digestion methane yield (Equation S3). For HRAS systems, we used kinetic model captures critical mechanisms including soluble substrate utilization, EPS production, adsorption-flocculation dynamics, and storage polymer formation (parameters in Tables S5-S8, Equation S4~S6), enabling facility-specific performance simulations[21]. Comparative effluent C/N ratio analyses from both processes provide essential guidance for technology selection based on wastewater characteristics. Together, these modeling tools offer robust evaluation of each system's capacity to meet the stringent C/N requirements essential for downstream autotrophic nitrogen removal processes.

$$SP_{CEPT} = SS_{in} - SS_{out} + k_{prec} \cdot D \quad \text{(Equation S3)}$$

$$COD_{out_{HRAS}}i = S_{BF_{i,t}} + S_{Bsi,t} + C_{Bi,t} + C_{Ui,t} + 0.4(X_{B_{i,t}} + X_{U_{i,t}}) \quad \text{(Equation S4)}$$

$$NH_3 - N_{outHRASi} = NH_3 - N_{ii,t} \quad \text{(Equation S5)}$$

$$TSS_{HRAS-sludgei} = (0.6 * \left(\frac{X_{Bi,t}}{i_{X_B}} + + \frac{X_{U_{i,t}}}{i_{X_U}}\right) + \frac{X_{EPS_{i,t}}}{i_{EPS}} + \frac{X_{STO_{i,t}}}{i_{STO}} + X_{OHO_{i,t}})/f_{\frac{VSS}{TSS}} \quad \text{(Equation S6)}$$

Where SP_{CEPT} is the sludge production from CEPT, SS_{in} and SS_{out} are suspended solids concentration in influent and effluent, K_{prec} is the sludge production coefficient, and D is the dosage of metal coagulant; $COD_{outHRAS}$ is the effluent COD of HRAS in the stable stage (time t), $NH_3 - N_{outHRAS}$ is the effluent ammonia nitrogen in the stable stage, and $TSS_{HRAS-sludge}$ is the

production of sludge of HRAS; assumption was made that 60% of particulate COD goes to sludge during the settling process after HRAS and 40% of it in effluent after settling process.

Table S2 Literature data of CEPT process performance and respective coagulant selection and dosage[16–20]

Meatal coagulant	Dosage	Influent COD	Influent TSS	Performance
**Alum with $FeCl_3$**	50 mg/L 20 mg/L	430~800	377~825	$COD\ removal(\%) = 153.33 - 0.01574 \times time - 0.00338 \times Q + 0.330142 \times T - 4.74168 \times pH + 0.02196 \times TDS - 0.02651 \times SS - 0.02337 \times COD$ $SS\ removal\% = 164.276 - 0.01905 \times time - 0.00295 \times Q + 0.2437 \times T - 5.44$[illegible] $\times pH + 0.01004 \times TDS - 0.0673 \times SS + 0.002039 \times COD$
**$FeCl_3$ Nalco polymer**	50 mg/L 0.5 mg/L	183~276	378~531	55%~87% COD removal, >90% TSS removal
**$FeCl_3$**	50 mg/L	222~822		76%~+-7 of COD
**Alum with polymer**	1.5 mg/L	263~748	60~204	79% COD removal and 92% TSS removal (large plant)
**Alum with polymer**	1.5 mg/L	404~584	76~376	75.5% COD removal and 90.1% TSS removal (small plant)
**$FeCl_3$**	50 mg/L	222~822	185~215	73% COD removal, 63% BOD removal, 30% TN removal
**$FeCl_3$**	40 mg/L	314	271	92.3% of TSS removal, 86.9% of COD removal, 77.4% BOD removal
**-**		-	-	60%~90% TSS removal, 40%~70% BOD removal

Table S3 Parameters used for empirical CEPT performance model in this study[19,20,22]

**Empirical model parameter**	**Value used in this study**
COD removal rate	75%
BOD removal rate	50%
NH_3-N removal rate	5%
TN removal rate	15%
TSS removal rate	90%
Sludge production coefficient due to coagulant	4~5 gSS/g Fe
Dosage of coagulant	25~50 mg/L Fe salt

Table S4 Modeled bioprocess components (organic substrate fractions and microbial products) in activated sludge systems[23,24]

Symbol	Description	Default fraction
$\boldsymbol{S_{Bs}}$	Rapidly biodegradable soluble organics	Min (BODinf, COD_inf*0.4)
$\boldsymbol{S_{Bf}}$	Slowly biodegradable soluble organics	0.25 of remaining
$\boldsymbol{C_B}$	Colloidal biodegradable organics	0.2
$\boldsymbol{C_U}$	Colloidal non-biodegradable organics	0.15
$\boldsymbol{X_B}$	Particulate biodegradable organics	0.3
$\boldsymbol{X_U}$	Particulate non-biodegradable organics	0.01
$\boldsymbol{X_{OHO}}$	Active ordinary heterotrophic organisms	-
$\boldsymbol{X_E}$	Particulate non-biodegradable endogenous products	-
$\boldsymbol{X_{EPS}}$	Extracellular polymeric substances	-
$\boldsymbol{X_{STO}}$	Intracellular storage polymeric substances	-

Table S5 Parameters used for kinetic HRAS performance model in this study[25]

Parameter	Value used in this study	Reference
HRT	30 mins	Ref 15~17
SRT	0.5 days	
DO	0.5 mg/L	
MLSS	2.5 g/L	
Default microbial kinetic parameters	See Table S6	
Particulate COD left in sludge	60%	
VSS/TSS	0.8	

Table S6 Default kinetic parameters for HRAS process modeling[21]

Symbol	Description	Value	Unit
$\boldsymbol{K_{B,HYD}}$	Saturation coefficient for X_B/X_{OHO}	0.03	g X_B/g X_{OHO}
$\boldsymbol{\mu_{OHO,Max}}$	Maximum growth rate of X_{OHO} on S_{Bf}	7.0	d^{-1}
$\boldsymbol{\mu^{a}_{OHO,Slow,Max}}$	Maximum growth rate of X_{OHO} on S_{Bs}	3.0	d^{-1}
$\boldsymbol{K_{Bf}}$	Half-saturation coefficient for S_{Bf} (X_{OHO})	5.0	g S_{Bf} m^{-3}
$\boldsymbol{K_{Bs}}$	Half-saturation coefficient for S_{Bs} (X_{OHO})	40	g S_{Bs} m^{-3}
$\boldsymbol{b_{OHO}}$	Decay rate for X_{OHO}	0.62	d^{-1}

$\boldsymbol{K_{O,OHO}}$	Half-saturation coefficient for S_{O2} (X_{OHO})	0.1	g S_{O2} m^{-3}
$\boldsymbol{q_{ADS}}$	Rate constant for adsorption	0.07	d^{-1}
$\boldsymbol{K_{SL}}$	Half-saturation coefficient for surface limitation	0.002	-
$\boldsymbol{q_{STO}}$	Rate constant for growth on X_{STO} (X_{OHO})	2	d^{-1}
$\boldsymbol{k_{EPS,MAX}}$	EPS formation coefficient	0.25	g COD_{EPS} $gVSS^{-1}$
$\boldsymbol{q_{EPS,HYD}}$	EPS hydrolysis	0.12	d^{-1}
$\boldsymbol{K_{EPS}}$	Half-saturation coefficient for EPS (X_{OHO})	50	g X_{EPS} m^{-3}
$\boldsymbol{q_{XB,HYD}}$	Particulate COD hydrolysis rate constant	3.5	d^{-1}
$\boldsymbol{k_{STO,MAX}}$	Maximum production yield for storage polymers	0.65	g X_{STO} gS_{Bf}^{-1}
$\boldsymbol{f_{shuny,MAX}}$	Fraction of X_{STO} in the active biomass	0.30	-
$\boldsymbol{q_{STO,HYD}}$	Storage hydrolysis rate constant	3.0	d^{-1}
$\boldsymbol{K_{STO,HYD}}$	Hydrolysis half-saturation coefficient for X_{STO} (X_{OHO})	0.15	g X_{STO} gX^{-1}_{OHO}
$\boldsymbol{K_{O_2,EPS}}$	Half-saturation coefficient for S_{O2}	1.5	g S_{O2} m^{-3}
$\boldsymbol{K_{O_2,STO}}$	Half-saturation coefficient STO for S_{O2}	1.0	g S_{O2} m^{-3}
$\boldsymbol{K_{EPS,HYD}}$	Saturation coefficient X_{EPS}	0.05	g X_{EPS} gX^{-1}_{OHO}

Table S7 Reaction rate equations for HRAS process model originated from ASM1 model [21]

**Reaction process**	**Reaction equation**
Aerobic growth of heterotrophs-fast	$r_1 = \mu_{OHO,\,max} \cdot \frac{S_{BF}}{K_{BF}+S_{BF}} \cdot \frac{S_{O_2}}{K_{O_2,OHO}+S_{O_2}} \cdot \frac{S_{NH_X}}{K_{NH_X,nut}+S_{NH_X}} \cdot X_{OHO}$
Aerobic growth of heterotrophs-slow	$r_{2a} = \mu_{OHO,\,slow,\,max} \cdot \frac{S_{Bs}}{K_{Bs}+S_{Bs}} \cdot \frac{K_{BF}}{K_{BF}+S_{BF}} \cdot \frac{S_{O_2}}{K_{O_2,OHO}+S_{O_2}} \cdot \frac{S_{NH_X}}{K_{NH_X,nut}+S_{NH_X}} \cdot X_{OHO}$
Aerobic growth of heterotrophs-slow	$r_{2b} = \mu_{OHO,\,max} \cdot \frac{S_{Bs}}{K_{Bs}+S_{Bs}} \cdot \frac{S_{O_2}}{K_{O_2,OHO}+S_{O_2}} \cdot \frac{S_{NH_X}}{K_{NH_X,nut}+S_{NH_X}} \cdot X_{OHO}$
Decay of heterotrophs	$r_3 = b_{OHO} \cdot X_{OHO,ACT}$
Hydrolysis of entrapped organics	$r_4 = q_{XB,HYD} \cdot \frac{X_B}{X_{OHO}} \cdot \frac{K_{B,HYD}+X_B}{X_{OHO}} \cdot \frac{S_{O_2}}{K_{O_2,OHO}+S_{O_2}} \cdot \frac{\eta_{HYD} \cdot K_{O_2,OHO}}{K_{O_2,OHO}+S_{O_2}} \cdot \frac{S_{NO_X}}{K_{NO_X}+S_{NO_X}} \cdot X_{OHO}$
Flocculation of colloidal substrate	$r_5 = q_{ADS} \cdot C_B \cdot (X_{OHO}+X_{ANO}) \cdot \left(\frac{K_{SL}}{\frac{C_B}{X_{OHO}+X_{ANO}}+K_{SL}}\right) \cdot \frac{X_{EPS}}{K_{EPS}+X_{EPS}}$

Flocculation of colloidal inserts	$r_6 = q_{ADS} \cdot C_U \cdot (X_{OHO} + X_{ANO}) \cdot (\frac{K_{SL}}{\frac{C_B}{X_{OHO} + X_{ANO}} + K_{SL}}) \cdot \frac{X_{EPS}}{K_{EPS} + X_{EPS}}$
Hydrolysis of storage products	$r_7 = q_{STO,HYD} \cdot \frac{\frac{X_{STO}}{X_{OHO}}}{K_{STO,HYD} + \frac{X_{STO}}{X_{OHO}}} \cdot \frac{K_{Bf}}{K_{Bf} + S_{Bf}} \cdot \frac{K_{Bs}}{K_{Bs} + S_{Bs}} \cdot \frac{S_{O_2}}{K_{O_2,OHO} + S_{O_2}} \cdot X_{OHO}$
EPS production	$r_8 = (\frac{K_{EPS,MAX}}{i_{CB}}) \times \left(\frac{S_{O_2}}{(K_{O,EPS} + S_{O_2})}\right)$
EPS hydrolysis	$r_8 = q_{EPA,HYD} \cdot X_{EPS}$
Storage products production	$r_{10} = (f_{shunt,MAX}) \times (S_{O_2}/(K_{O,STO} + S_{O_2})$

Table S8 Stoichiometric and conversion parameters for organic fractions in activated sludge modeling[23,26]

**Parameter**	**Value used in this study**
$\boldsymbol{f_{\frac{VSS}{TSS}}}$	0.85
$\boldsymbol{i_{XB}}$	1.48 gCOD/gVSS
$\boldsymbol{i_{XU}}$	1.8 gCOD/gTSS
$\boldsymbol{i_{XEPS}}$	1.6
$\boldsymbol{i_{XSTO}}$	1.42
$\boldsymbol{f_{U}}$	0.08

Part II: Compatibility framework for CEPT-PdN/A and HRAS-PN/A, and energy balance modeling

The assessment of compatible facilities for implementing either Pathway I or Pathway II was based on the effluent C/N ratios achieved through CEPT and HRAS process modeling[14,15]. The distribution of COD/TN ratios from CEPT and COD/NH3-N ratios from HRAS is illustrated in Figures S7 and S8. A key focus of this evaluation was the changes in energy consumption and energy recovery. For comprehensive energy balance modeling of the integrated CEPT-PdN/A and HRAS-PN/A systems, we meticulously accounted for all relevant energy fluxes within the system boundaries. While CEPT and HRAS processes redistribute more COD to wasted sludge, thereby enhancing biogas potential, they also may require additional electricity and heat for primary treatment and sludge heating. However, the subsequent PdN/A and PN/A processes significantly reduce aeration demands, which is particularly beneficial since aeration typically accounts for over 60% of total electricity use in conventional treatment facilities. In our modeling approach, we allocated 60% of the original EUI to aeration and 40% to other functions, including pumping and miscellaneous operations (sensitivity analysis was conducted for this assumption). The complete energy consumption profiles for both pathways, covering both electricity and thermal energy

requirements, are formally presented in Equations S7 to S10. A summary of all detailed parameters can be found in Table S9. If a facility is compatible with both pathways, the one with higher energy self-sufficiency potential is selected.

$$ED_{CEPT-PdN/A_{ele,i}} = EUI_{CEPT} \cdot Q_i + Q_i \cdot \alpha \cdot EUI_i + \gamma \cdot Q_i \cdot \beta \cdot EUI_i + Y_{CEPT\,i} \cdot e_{dewater}$$

(Equation S7)

$$ED_{CEPT-PdN/A_{heat,i}} = (1 + R_{loss}) \cdot \frac{Y_{CEPT\,i}}{c_{solid}} \cdot C_{sludge} \cdot \frac{HDD_{meso\,i}}{3600}$$ (Equation S8)

$$ED_{HRAS-PN/A_{ele,i}} = EUI_{HRAS} \cdot Q_i + Q_i \cdot \alpha \cdot EUI_i + \gamma \cdot Q_i \cdot \beta \cdot EUI_i \cdot (1 - \varphi) + Y_{HRAS\,i} \cdot e_{dewater}$$

(Equation S9)

$$ED_{HRAS-PN/A_{heat,i}} = (1 + R_{loss}) \cdot \frac{Y_{HRAS\,i}}{c_{solid}} \cdot C_{sludge} \cdot \frac{HDD_{meso\,i}}{3600}$$ (Equation S10)

Where $ED_{CEPT-PdN/A_{ele,i}}$ is the electricity demand of pathway I in facility i, $ED_{HRAS-PN/A_{ele,i}}$ is the heat demand of pathway I in facility i; $ED_{HRAS-PN/A_{ele,i}}$ is the electricity demand of pathway II in facility i, and $ED_{HRAS-PN/A_{heat,i}}$ is the heat demand of pathway II in facility i.

Table S9 Key parameters used in energy balance modeling for CEPT-PdN/A and HRAS-PN/A processes[4,27]

Parameter symbol	Description	Value
c_{solid}	Solid content in CEPT & HRAS sludge (total solids)	20%
EUI_{CEPT}	Electricity use intensity for CEPT (mixing/stirring)	0.03 kWh/m^3
EUI_{HRAS}	Electricity use intensity for HRAS (aeration + recirculation + mixing)	0.162 kWh/m^3
α	Fraction of electricity for non-aeration processes (mixing, pumping)	40%
β	Fraction of electricity for aeration	60%
γ	Flow ratio directed to aeration zones	50%
φ	Aeration saving factor due to partial nitrification ($NH_4^+ \rightarrow NO_2^-$ vs. $NH_4^+ \rightarrow NO_3^-$)	60%
Y_{CEPT} , Y_{HRAS}	Wasted sludge yield from CEPT or HRAS	Modeled

Uncertainty characterization and sensitivity assessment in the modeling approach

Stochastic frontier analysis (SFA) for technical inefficiency assessment

Given that the efficiency scores derived from the SFA model play a crucial role in assessing the operational efficiency of facilities, ensuring robustness is essential. To evaluate the robustness of our SFA model for energy use efficiency, we conducted both uncertainty analysis and sensitivity analysis. We performed uncertainty testing using the Bootstrap method, resampling (with replacement) facilities from the national dataset (resampling time = 500). For each iteration, we recorded the distribution of efficiency scores (focusing on mean and standard deviation) and calculated the coefficient of variation (CV) across all 500 resamples. A CV below 10% was considered acceptable. Additionally, we examined the stability of the SFA regression parameters—including the standard errors of the inefficiency term, random error term, and regression coefficients—by assessing their 95% confidence interval. To assess SFA sensitivity, we first included all potential independent variables (COD, BOD, TN, NH3-N, SS, TP, and flow rate) and observed their impact on the model. We then systematically reduced the number of predictors to mitigate multicollinearity, identifying the most influential variables while maintaining model simplicity. Furthermore, we compared two functional forms for the cost function—Cobb-Douglas and translog—to determine the best fit. Finally, we evaluated the influence of outliers on both the regression results and the predicted efficiency scores. To validate our SFA-derived efficiency estimates, we compared them with a previous Chines national-wide energy efficiency study using DEA, which reported significantly lower mean efficiency scores (0.0163) suggesting greater inefficiencies[28]. This discrepancy arises from DEA's methodological limitation of being unable to distinguish between random statistical noise and true technical inefficiency, thereby overestimating operational shortcomings by attributing all deviations to technical inefficiency. In contrast, our SFA approach explicitly separates random variability (e.g., measurement errors) from technical inefficiency, yielding more accurate estimates that better reflect true operational performance, thus explaining why our results show higher efficiency scores than the DEA benchmark.

Sensitivity assessment for the CEPT and HRAS process modeling

Robust process modeling of CEPT and HRAS is essential to evaluate whether their effluents meet the stringent requirements for downstream mainstream Anammox processes, whether PdN/A or PN/A. The reliability of these models directly impacts pathway selection and energy balance calculations. For CEPT, an empirical process model was adopted due to its physicochemical simplicity, with key performance parameters derived from literature. Sensitivity analyses were conducted by varying removal rates of COD, BOD, TN, and SS by ±5% to assess process robustness. Given that coagulant dosage influences COD redistribution and sludge production,

thereby affecting energy recovery and consumption, additional sensitivity tests were performed on coagulant dose, sludge yield coefficients, and energy use intensity for mixing (±10% of default values). For HRAS process modeling, operational parameters were systematically evaluated despite being sourced from comprehensive literature reviews. Key variables, including hydraulic retention time (HRT), solids retention time (SRT), and influent fast-soluble biodegradable fraction, were tested at ±50% and ±5% deviations, respectively. These assessments focused on critical outputs: effluent C/N ratio, sludge production, and COD redistributed into wasted sludge. A nominal influent composition (COD = 500 mg/L, BOD = 300 mg/L, NH_3-N = 40 mg/L) served as the baseline for HRAS sensitivity analysis.

Sensitivity assessment for energy balance modeling

Sensitivity analysis for the energy balance model evaluated both consumption (electricity/heat) and recovery (methane production) components. For energy consumption, key parameters included sludge dewatering electricity intensity (tested across 5–20 kWh/ton dry sludge), sludge solid content (±10% variation), and process-specific electricity use intensities for CEPT and HRAS (±10% adjustment). Energy recovery was modeled via a hybrid kinetic approach for anaerobic digestion, with sensitivity tests on three critical variables: waste utilization rate, biomass decay rate, and biomass yield coefficient—each spanning literature-reported lower to upper bounds to assess their influence on methane output.

SUPPLEMENTARY RESULTS

Decomposition of regional energy performance

This study systematically investigated the underlying causes of regional disparities in wastewater treatment energy performance by evaluating whether observed variations primarily stem from inherent wastewater characteristics (e.g., high carbon and nitrogen concentrations requiring greater energy inputs for compliance) or other operational inefficiencies. Through comprehensive analysis of facility-level energy use intensity across multiple variables, including treatment scale, secondary treatment processes, local climate conditions (quantified via annual heating degree days for mesophilic anaerobic digestion), effluent discharge standards, and detailed influent characteristics (COD, BOD, TN, NH3-N, SS, and TP), we identified significant correlations exclusively between EUI with treatment scale and influent composition (Figures S3 and S4). Subsequent principal component analysis coupled with K mean clustering grouped regions by similar flow scale influent profiles, yet revealed persistent EUI differences within clusters, strongly suggesting that internal operational inefficiencies (beyond scale and wastewater quality parameters) constitute a major contributor to regional energy performance disparities.

Table S10 Variance decomposition of energy use intensity (ANOVA test-based approach)

EUI	Total SS	Percentage of between-group variation	Percentage of within-group variation
Flow based EUI (kWh/m^3)	0.1	48%	52%
Pollutant removal based EUI (kWh/ kg COD removed)	2.9	28%	72%

Stochastic frontier model specification

Following a comprehensive evaluation of both Cobb-Douglas and translog functional forms, the Cobb-Douglas cost function was selected for our SFA based on its superior parameter significance. Technical inefficiency was modeled using a half-normal distribution, consistent with conventional econometric approaches for efficiency estimation.

Table S11 Estimated parameters of the stochastic frontier cost function

Parameter		Value	Statistical significance
Cost frontier intercept	β_o	4.11	p< 0.05 (Significant)
Treatment scale	β_1	-0.11	

Influent COD	β_2	-1.06	
**Influent BOD**	β_3	0.032	
**Influent SS**	β_4	0.045	
**Influent TP**	β_5	0.045	
**Effluent COD**	β_6	0.049	
**Influent Nh3-N**	β_7	0.0046	
**Influent TN**	β_8	0.143	
Standard deviation (technical inefficiency)	σ_u	1.21	
Standard deviation (random noise)	σ_v	0.33	
Inefficiency-to-noise ratio	$\lambda = \frac{\sigma_u}{\sigma_v}$	6.01	

Model uncertainty and sensitivity assessment results

Table S12 Robustness assessment of stochastic frontier model parameters

Indicator	Methodology	Result
Efficiency score stability: coefficient of variation (mean efficiency)	Bootstrap resampling (n = 500)	2.4%
Efficiency score stability: coefficient of variation (SD of efficiency)		3.7%
95% confidence interval of $\boldsymbol{\beta_0}$		[3.56, 4.65]
95% confidence interval of $\boldsymbol{\beta_1}$		[-0.14, -0.082]
95% confidence interval of $\boldsymbol{\beta_2}$		[-1.241, -0.88]
95% confidence interval of $\boldsymbol{\beta_3}$		[-0.057, 0.114]
95% confidence interval of $\boldsymbol{\beta_4}$		[-0.032, 0.121]
95% confidence interval of $\boldsymbol{\beta_5}$		[-0.041, 0.123]
95% confidence interval of $\boldsymbol{\beta_6}$		[-0.046, 0.146]
95% confidence interval of $\boldsymbol{\beta_7}$		[-0.122, 0.135]
95% confidence interval of $\boldsymbol{\beta_8}$		[0.0058, 0.313]
95% confidence interval of $\boldsymbol{v}$		[0.27, 0.39]
95% confidence interval of $\boldsymbol{u}$		[1.34, 1.59]
Outlier sensitivity: Spearman's correlation (vs. original data)	Gradual outlier removal procedure	0.1%

Table S13 Sensitivity analysis of anaerobic digestion energy recovery parameters

Parameter	Symbol	Test range	Output variation (change on methane yield)
Waste utilization rate	E	0.6~0.9	40%

Biomass decay rate	k_d	0.02~0.4 d^{-1}	4.4%
Sludge yield coefficient	Y	0.04~1 g VSS/g BOD	5.7%

Table S14 Sensitivity analysis of energy consumption parameters

Parameter	Test range	Energy consumption variation
Sludge dewatering electricity use intensity	5–20 kWh/ton DS	0.54%
Sludge solid content	±10%	0.072%

Table S15 Sensitivity analysis of CEPT performance and its energy balance parameters

Category	Parameter	Variation range	Output metric	Impact variation
Removal efficiency	COD removal rate	+-5%	Effluent C/N ratio	16%
	TN removal rate	+-5%	Effluent C/N ratio	5.9%
	BOD removal rate	+-5%	Biogas production	11%
	SS removal rate	+-5%	Energy consumption	0.11%
Operational control	Coagulant dosage	+-10%		0.17%
	Sludge production coefficient due to coagulant	+-10%		0.17%
	CEPT EUI	+-10%		0.45%

Table S16 Sensitivity analysis of HRAS process performance to operational parameters

Operational parameter	Variation	Effect on C/N ratio	Effect on sludge production	Effect on sludge COD content
Hydraulic retention time	×2	+1.29%	+14.8%	+14.7%
	÷2	+3.86%	-16.2%	-16.2%
Sludge retention time	×2	-	-50%	-50%
	÷2	-	+100%	+100%
Soluble biodegradable COD fraction	+0.5%	-11.2%	-5.78%	-5.81%
	-0.5%	+12.0%	+5.44%	+5.41%
Dissolved oxygen	+5%	-0.17%	-0.13%	-0.31%
	-5%	+0.47%	+0.18%	+0.17%

*(Baseline conditions : HRT = 30 min, SRT = 0.5 d, Soluble biodegradable COD fraction = 40%, DO = 0.5 mg/L)

Table S17 Sensitivity analysis of key parameters on HRAS process Energy consumption

Parameter	Variation	Energy consumption variation
HRAS Energy Use Intensity (EUI)	±20%	4.2%
Aeration/non-aeration Energy Ratio in PN/A	±20%	1.2%
HRAS sludge production (from kinetic modeling)	±50%	2%

*(Baseline: EUI_{HRAS} = 0.162 kWh/m³, Ratio of energy used for aeration = 60%, Sludge solid content = 20%)

Table S18 Coefficients of variation (%) for influent characteristics and flow scale across K-means clusters

Cluster	COD	BOD	TN	NH3-N	SS	Flow scale
1	2.8%	4.6%	7.9%	7.4%	2.4%	3.7%
2	3.0%	5.9%	3.8%	6.0%	10.7%	3.0%
3	1.3%	2.3%	4.6%	4.5%	6.6%	2.1%
4	7.3%	3.5%	13%	12%	14.6%	12%
5	7.9%	2.9%	1.3%	2.5%	3.3%	7.5%
6	3.9%	6.0%	2.0%	4.3%	4.7%	12%

Table S19 China's Provincial-Level Acronyms

Full name	Acronym	Full name	Acronym	Full name	Acronym
**Beijing**	BJ	Heilongjiang	HLJ	Shanxi	SX
**Chongqing**	CQ	Henan	HEN	Sichuan	SC
**Shanghai**	SH	Hubei	HUB	Yunnan	YN
**Tianjin**	TJ	Hunan	HUN	Zhejiang	ZJ
**Anhui**	AH	Jiangsu	JS	Guangxi	GX
**Fujian**	FJ	Jiangxi	JX	Inner Mongolia	IM
**Gansu**	GS	Jilin	JL	Ningxia	NX
**Guangdong**	GD	Liaoning	LN	Xizang	XZ
**Guizhou**	GZ	Qinghai	QH	Xinjiang	JX
**Hainan**	HAN	Shaanxi	SNX		
**Hebei**	HEB	Shandong	SD		

* This work excludes the Hong Kong SAR, Macao SAR, and Ningxia Hui because WWTP data for these areas were not available.

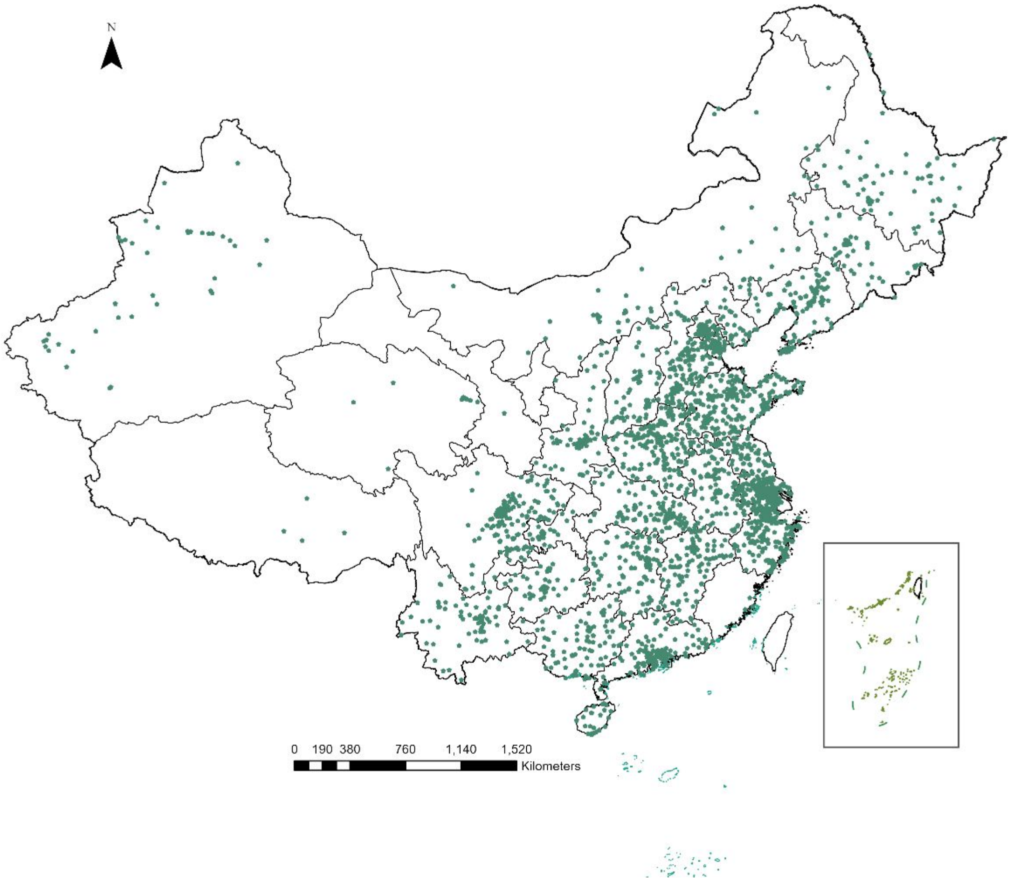

Figure S1 Geographical distribution of wastewater treatment facilities in China (n = 2,961) with valid information were identified in this study, and no data in Ningxia Autonomous Region.

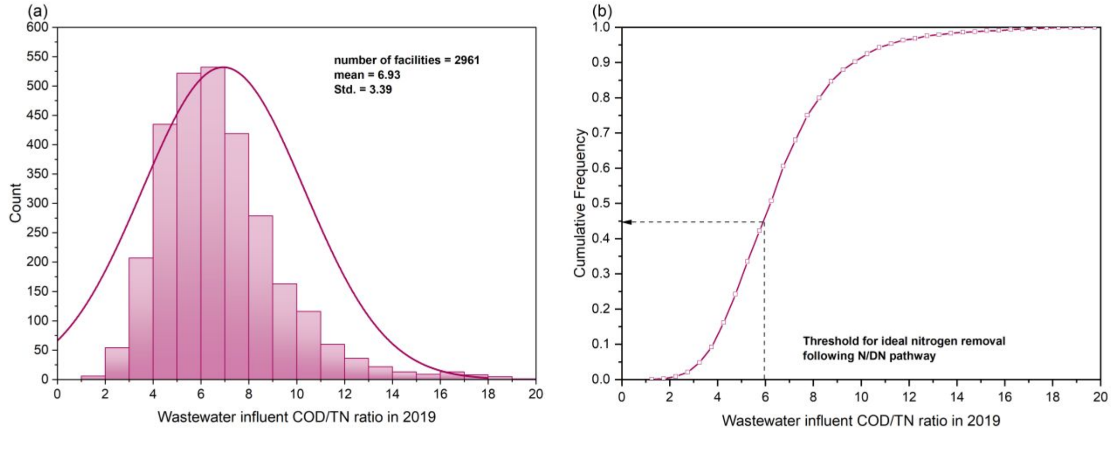

Figure S2 National wastewater influent characteristics in 2019 (n=2,961 facilities): (a) COD/TN ratio distribution and (b) cumulative frequency distribution

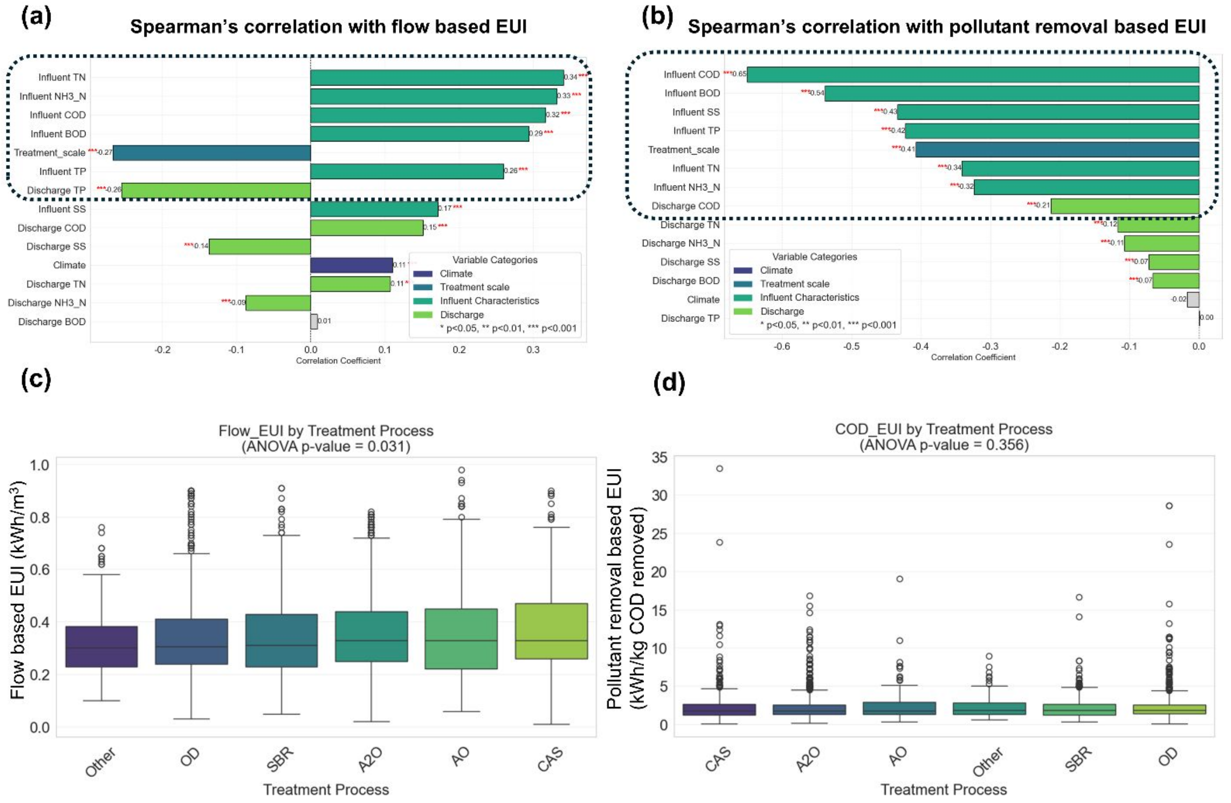

Figure S3 Correlation and ANOVA analysis of energy use intensity metrics. (a-b) Spearman's correlation coefficients between EUI measures (flow-based and pollutant removal-based) and external factors, grouped by climate conditions, treatment scale, influent characteristics, and discharge requirements (|r| > 0.3 considered practically significant). (c-d) Variation in EUI across treatment processes, showing ANOVA results (while flow EUI demonstrated significant p-values, post-hoc Tukey HSD tests revealed no significant pairwise differences between specific treatment processes).

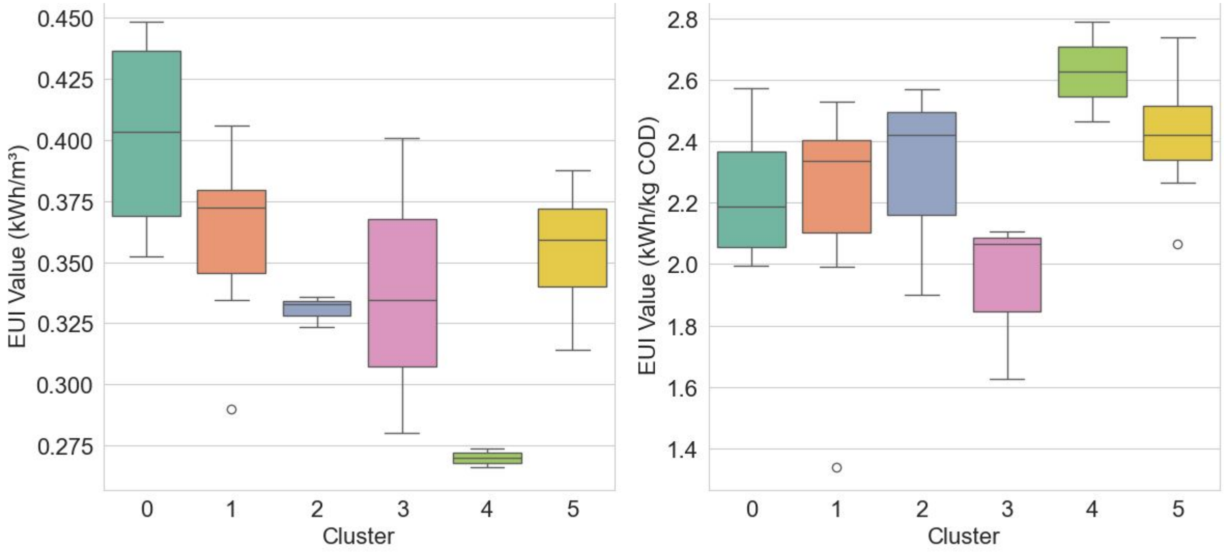

Figure S4 Distribution of flow EUI (left) and pollutant removal EUI (right) in different clusters

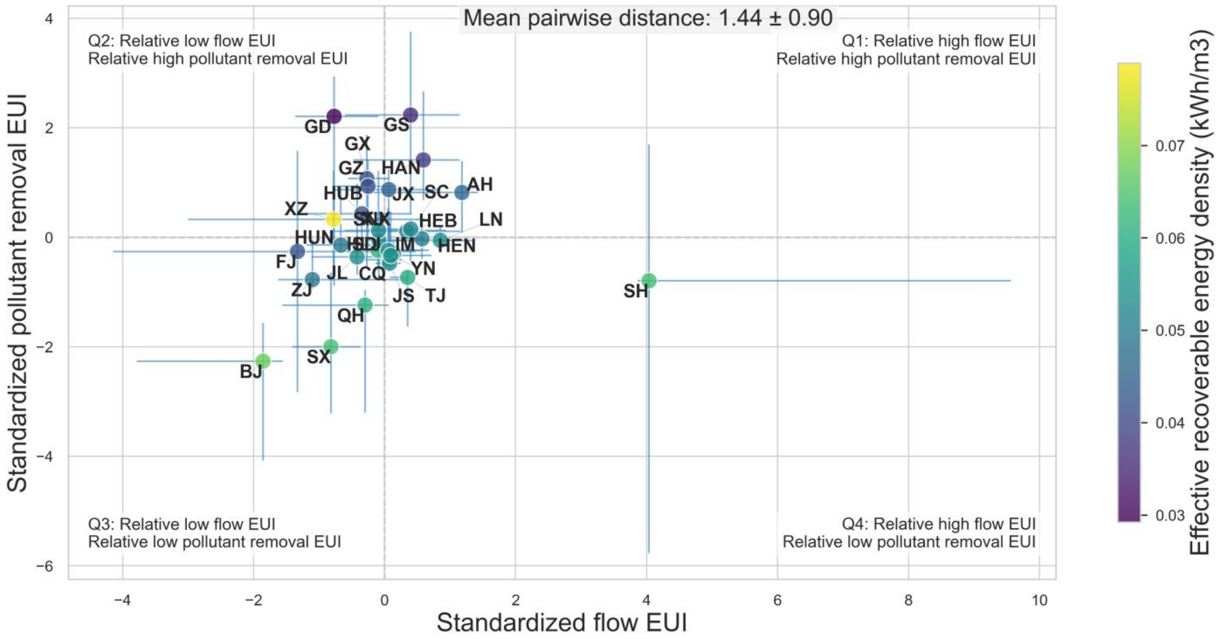

Figure S5 Regional energy performance disparities following system optimization with anaerobic digestion (AD) and combined heat and power (CHP) energy recovery

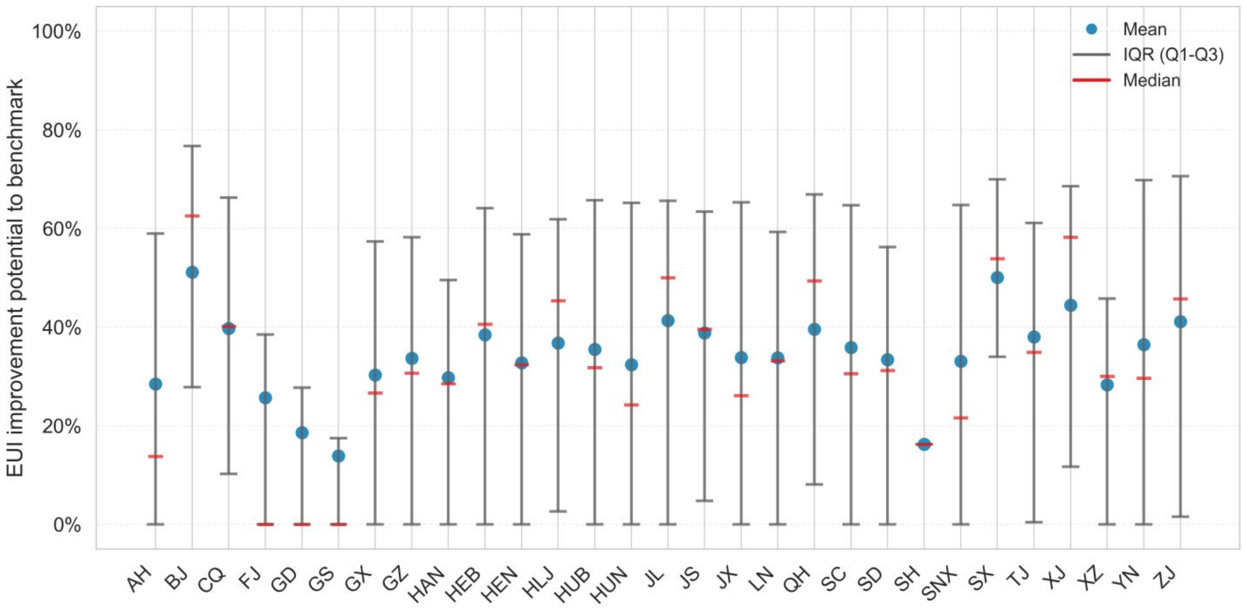

Figure S6 Provincial-level energy use performance gaps relative to SFA benchmark targets

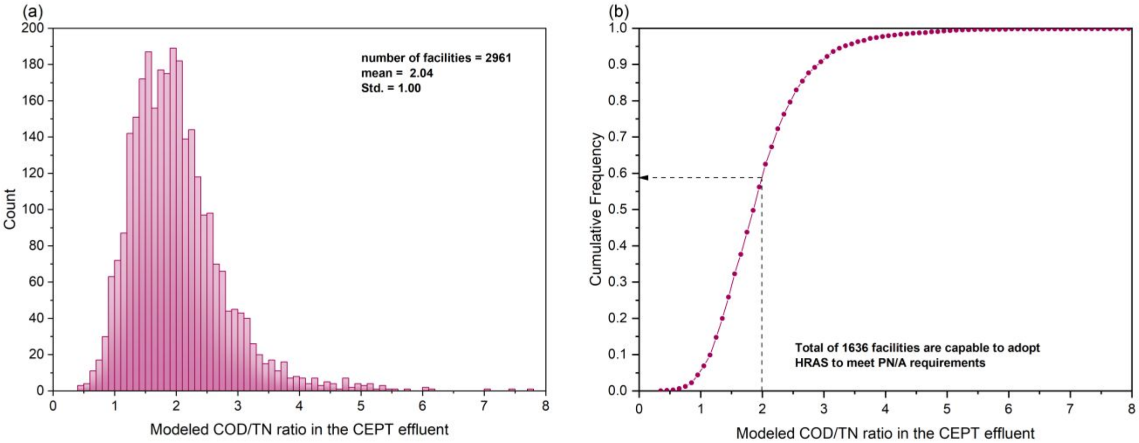

Figure S7 Effluent COD/TN ratio distribution from CEPT process modeling: (a) frequency of COD/TN ratios (mean = 2.04 ± 1.00 SD) across Chinese WWTPs. (b) cumulative distribution showing 1636 facilities achieve ratios <2.0 post-HRAS

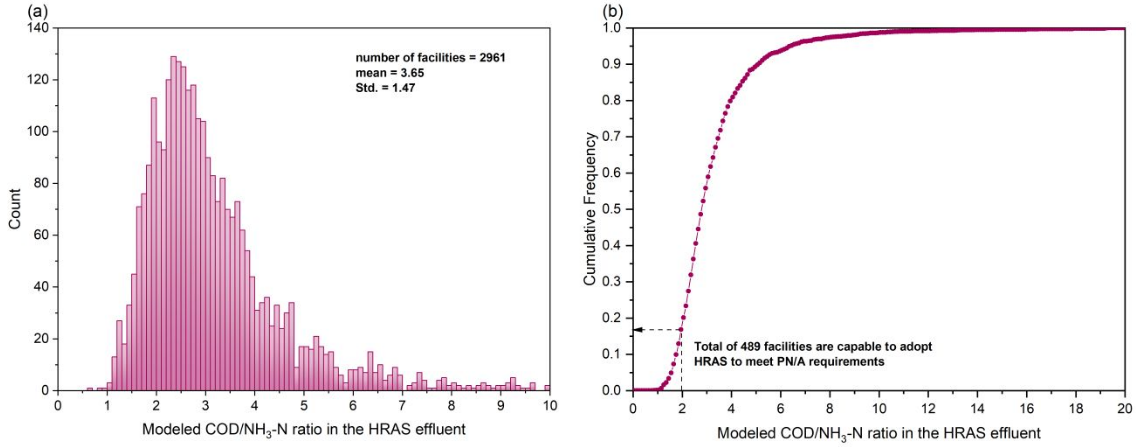

Figure S8 Effluent COD/NH_3-N ratio distribution from HRAS process modeling: (a) frequency of COD/NH_3-N ratios (mean = 3.65 ± 1.47 SD) across Chinese WWTPs. (b) cumulative distribution showing 489 facilities achieve ratios <2.0 post-HRAS.

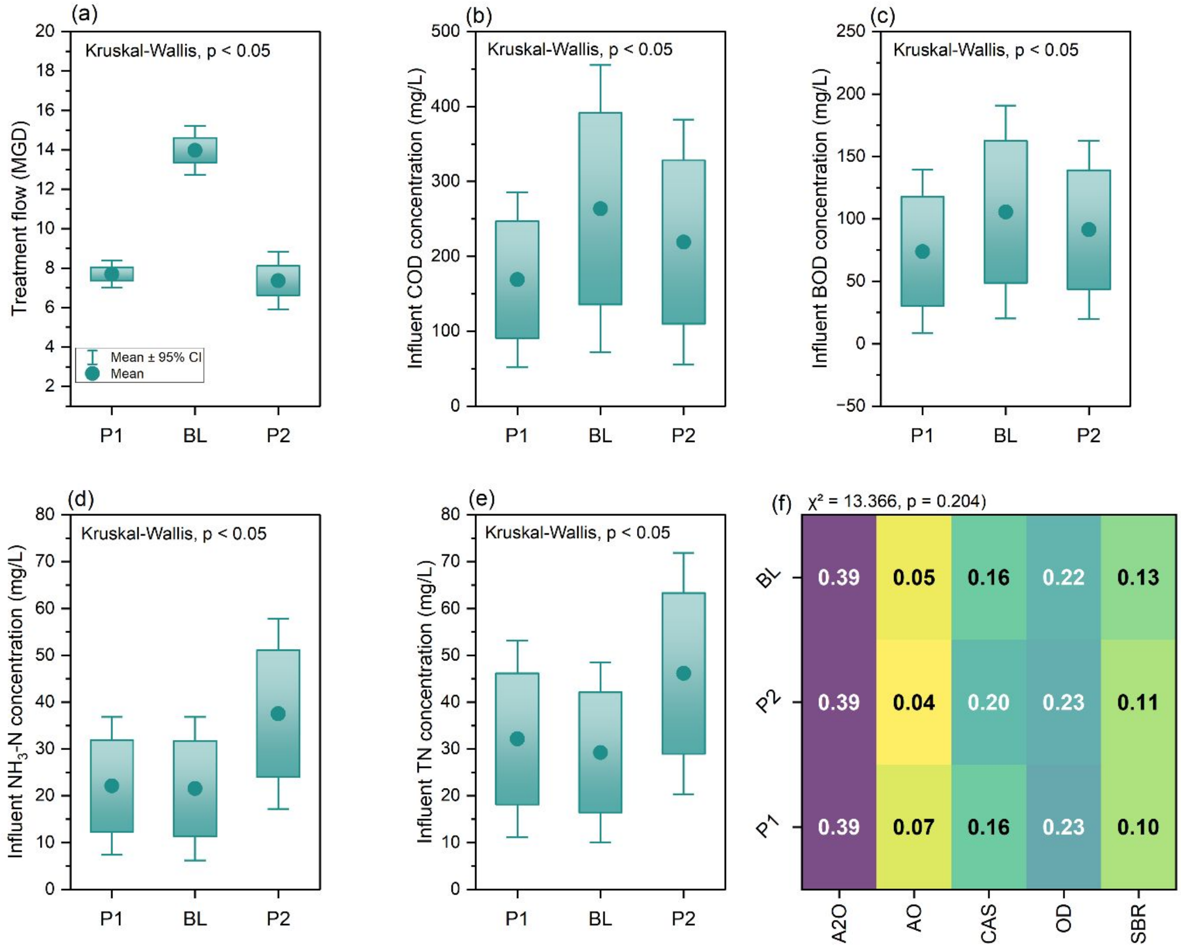

Figure S9 Characterization of selected wastewater treatment pathways (baseline, pathway I, and pathway II): (a-e) distribution of (a) flow scale, (b) influent COD, (c) influent BOD, (d) influent NH_3-N, and (e) influent TN across pathways, with significant differences tested by Kruskal-Wallis ($p<0.05$). (f) association between the selected pathways and conventional treatment technologies.

REFERENCES

1. *Municipal Wastewater Treatment Energy Use Standards*. (2021).
2. Dai, X., Hou, L., Zhang, L., Zhang, L. & Yang, D. Safe Disposal and Resource Recovery of Urban Sewage Sludge in China. *Chinese Journal of Engineering Science* 24, 145 (2022).
3. Cao, Y., Van Loosdrecht, M. C. M. & Daigger, G. T. The bottlenecks and causes, and potential solutions for municipal sewage treatment in China. *Water Pract Technol* 15, 160–169 (2020).
4. Maktabifard, M., Zaborowska, E. & Makinia, J. Achieving energy neutrality in wastewater treatment plants through energy savings and enhancing renewable energy production. *Reviews in Environmental Science and Biotechnology* vol. 17 655–689 Preprint at https://doi.org/10.1007/s11157-018-9478-x (2018).
5. Chang, H., Zhao, Y., Xu, A., Damgaard, A. & Christensen, T. H. Mini-review of inventory data for the dewatering and drying of sewage sludge. *Waste Management and Research* vol. 41 1081–1088 Preprint at https://doi.org/10.1177/0734242X221139170 (2023).
6. Appels, L., Baeyens, J., Degrève, J. & Dewil, R. Principles and potential of the anaerobic digestion of waste-activated sludge. *Progress in Energy and Combustion Science* vol. 34 755–781 Preprint at https://doi.org/10.1016/j.pecs.2008.06.002 (2008).
7. Garkoti, P., Ni, J. Q. & Thengane, S. K. Energy management for maintaining anaerobic digestion temperature in biogas plants. *Renewable and Sustainable Energy Reviews* vol. 199 Preprint at https://doi.org/10.1016/j.rser.2024.114430 (2024).
8. States Department of Energy, U. *Combined Heat and Power Technology Fact Sheet Series Overview of CHP Technologies*.
9. Xiong, Y. T. *et al.* Geographic distribution of net-zero energy wastewater treatment in China. *Renewable and Sustainable Energy Reviews* 150, (2021).
10. Heat Values of Various Fuels. *https://world-nuclear.org/information-library/facts-and-figures/heat-values-of-various-fuels*.
11. Zhang, M. *et al.* C/N ratios inform sustainable aerobic denitrification for nitrogen pollution control: Insights into the key parameter from a view of metabolic division. *J Clean Prod* 414, 137565 (2023).
12. Cao, Y., van Loosdrecht, M. C. M. & Daigger, G. T. Mainstream partial nitritation–anammox in municipal wastewater treatment: status, bottlenecks, and further studies.

Applied Microbiology and Biotechnology vol. 101 1365–1383 Preprint at https://doi.org/10.1007/s00253-016-8058-7 (2017).

13. Cogert, K. I., Ziels, R. M. & Winkler, M. K. H. Reducing Cost and Environmental Impact of Wastewater Treatment with Denitrifying Methanotrophs, Anammox, and Mainstream Anaerobic Treatment. *Environ Sci Technol* 53, 12935–12944 (2019).

14. Miao, Y. *et al.* Partial nitrification-anammox (PNA) treating sewage with intermittent aeration mode: Effect of influent C/N ratios. *Chemical Engineering Journal* 334, 664–672 (2018).

15. Sun, Y. *et al.* Influence of C/N ratio and ammonia on nitrogen removal and N2O emissions from one-stage partial denitrification coupled with anammox. *Chemosphere* 341, (2023).

16. Yu, I. W. *Bench-Scale Study of Chemically Enhanced Primary Treatment in Brazil Signature of the Author Department of Civil and Environme# Engineering*.

17. Abdel-Fatah, M. A., Al Bazedi, G. A., Hawash, S. I. & Amin, A. Feasibility Study on Wastewater Treatment Using Chemically Enhanced Primary Treatment. *Letters in Applied NanoBioScience* 11, 3917–3926 (2022).

18. Al Bazedi, G. A. & Abdel-Fatah, M. A. Correlation between operating parameters and removal efficiency for chemically enhanced primary treatment system of wastewater. *Bull Natl Res Cent* 44, (2020).

19. Aiyuk, S., Amoako, J., Raskin, L., Van Haandel, A. & Verstraete, W. Removal of carbon and nutrients from domestic wastewater using a low investment, integrated treatment concept. *Water Res* 38, 3031–3042 (2004).

20. Finger, D. & Melcer, H. *OPERATOR ESSENTIALS What Every Operator Needs to Know About Chemically Enhanced Primary Treatment*.

21. Bf, N. S., Bs C B C U X B X U, S., Oho, X. & Eps, A. X. *Modeling of Organic Substrate Transformation in the High-Rate Activated Sludge Process APPENDIX Table A1 | Partial Gujer Matrix Processes and Stoichiometric Coefficients for the HRAS Model*. (2015).

22. Shewa, W. A. & Dagnew, M. Revisiting chemically enhanced primary treatment of wastewater: A review. *Sustainability (Switzerland)* vol. 12 Preprint at https://doi.org/10.3390/SU12155928 (2020).

23. Henze, M., Gujer, W., Mino, T. & van Loosedrecht, M. Activated Sludge Models ASM1, ASM2, ASM2d and ASM3. *Water Intelligence Online* 5, 9781780402369–9781780402369 (2015).

24. Rieger, L. *et al. THE EAWAG BIO-P MODULE FOR ACTIVATED SLUDGE MODEL NO. 3*. *Wat. Res* vol. 35 (2001).

25. Guthi, R. S. *et al.* A-Stage process – Challenges and drawbacks from lab to full scale studies: A review. *Water Research* vol. 226 Preprint at https://doi.org/10.1016/j.watres.2022.119044 (2022).

26. Tchobanoglous, George. *et al. Wastewater Engineering: Treatment and Resource Recovery*. (McGraw-Hill Education, 2014).

27. Lin, C., Xiao, X., Li, Y. Y. & Liu, J. Evaluation of the economic and environmental benefits of partial nitritation anammox and partial denitrification anammox coupling preliminary treatment in mainstream wastewater treatment. *Renewable and Sustainable Energy Reviews* 188, (2023).

28. Yang, J. & Chen, B. Energy efficiency evaluation of wastewater treatment plants (WWTPs) based on data envelopment analysis. *Appl Energy* 289, (2021).
